# Genome-wide identification, structure characterization, and expression pattern profiling of aquaporin gene family in cucumber

**DOI:** 10.1186/s12870-019-1953-1

**Published:** 2019-08-07

**Authors:** Yong-Xing Zhu, Lei Yang, Ning Liu, Jie Yang, Xiao-Kang Zhou, Yu-Chen Xia, Yang He, Yi-Qin He, Hai-Jun Gong, Dong-Fang Ma, Jun-Liang Yin

**Affiliations:** 1 Hubei Key Laboratory of Waterlogging Disaster and Agricultural Use of Wetland/College of Horticulture and Gardening, Yangtze University, Jingzhou, 434000 Hubei China; 2 College of Horticulture, Northwest A and F University, Yangling, 712100 Shaanxi China

**Keywords:** Phylogenetic analysis, Gene structure, Protein characterization, RNA-seq, Abiotic stresses, Water loss rate

## Abstract

**Background:**

Aquaporin (AQP) proteins comprise a group of membrane intrinsic proteins (MIPs) that are responsible for transporting water and other small molecules, which is crucial for plant survival under stress conditions including salt stress. Despite the vital role of AQPs, little is known about them in cucumber (*Cucumis sativus* L.).

**Results:**

In this study, we identified 39 aquaporin-encoding genes in cucumber that were separated by phylogenetic analysis into five sub-families (PIP, TIP, NIP, SIP, and XIP). Their substrate specificity was then assessed based on key amino acid residues such as the aromatic/Arginine (ar/R) selectivity filter, Froger’s positions, and specificity-determining positions. The putative *cis*-regulatory motifs available in the promoter region of each AQP gene were analyzed and results revealed that their promoter regions contain many abiotic related *cis*-regulatory elements. Furthermore, analysis of previously released RNA-seq data revealed tissue- and treatment-specific expression patterns of cucumber AQP genes (CsAQPs). Three aquaporins (*CsTIP1;1*, *CsPIP2;4*, and *CsPIP1;2*) were the most transcript abundance genes, with *CsTIP1;1* showing the highest expression levels among all aquaporins. Subcellular localization analysis in *Nicotiana benthamiana* epidermal cells revealed the diverse and broad array of sub-cellular localizations of CsAQPs. We then performed RNA-seq to identify the expression pattern of CsAQPs under salt stress and found a general decreased expression level of root CsAQPs. Moreover, qRT-PCR revealed rapid changes in the expression levels of CsAQPs in response to diverse abiotic stresses including salt, polyethylene glycol (PEG)-6000, heat, and chilling stresses. Additionally, transient expression of AQPs in *N*. *benthamiana* increased leaf water loss rate, suggesting their potential roles in the regulation of plant water status under stress conditions.

**Conclusions:**

Our results indicated that CsAQPs play important roles in response to salt stress. The genome-wide identification and primary function characterization of cucumber aquaporins provides insight to elucidate the complexity of the AQP gene family and their biological functions in cucumber.

**Electronic supplementary material:**

The online version of this article (10.1186/s12870-019-1953-1) contains supplementary material, which is available to authorized users.

## Background

Salt stress is one of the major environmental constraints that limit crop growth and cause significant yield loss in large areas throughout the world [1]. It has been estimated that 45 million hectares of irrigated land are affected by salt stress and this situation is expected to increase due to global climate changes and as a results of intensive irrigation practices. Commonly, salt stress decreases plant root water uptake due to both osmotic and toxic effects, depending on the salt concentration present [2, 3]. Water transport through plant tissues or from the xylem and phloem may occur by three different pathways: (1) the apoplastic path around the protoplasts; (2) the symplastic path through the plasmodesmata; and (3) the transcellular path across the cell membranes [4]. During responses of plants to adverse stresses (e.g. salt and drought), water transmembrane transport constitutes an important regulatory pathway [2]. Aquaporins (AQPs) are a family of small (21 to 34 kDa) channel-forming transmembrane proteins that belong to the membrane intrinsic proteins (MIPs) family. They have been shown to act as multifunctional channels that transport water and many small molecules such as ammonia (NH_3_), carbon dioxide (CO_2_), nitric oxide (NO), formamide, glycerol, hydrogen peroxide (H_2_O_2_), and metalloids such as silicon and boron [5].

Generally, AQPs are highly conserved in all living organisms, consisting of six transmembrane helices (TMHs) connected by five loops (A to E), and cytosolic N- and C-termini [6]. Loops B (cytosolic) and E (non-cytosolic) contain the highly conserved NPA (Asparagine-Proline-Alanine) boxes, and make up helices that fold back into the core of the protein to form one of the two major constrictions of the pore, the NPA region [5]. A second filter region is the aromatic/Arginine (ar/R) constriction located at the non-cytosolic end of the pore. Substrate selectivity of AQPs is controlled by the amino acid residues of the NPA and ar/R filters, as well as other parts of the channel [5].

On the basis of subcellular localization and sequence homology, AQPs can be divided into five evolutionarily distinct subfamilies in plant, which include the plasma membrane intrinsic proteins (PIPs), the tonoplast intrinsic proteins (TIPs), the Nodulin-26-like intrinsic proteins (NIPs), the small basic intrinsic proteins (SIPs), and the X intrinsic proteins (XIPs) [6]. The PIP subfamily can be further subdivided into PIP1 and PIP2 by the distinction of the lengths of their amino and carboxyl termini, with the amino termini of the PIP1 being longer than that of the PIP2 [7].

The plant AQP protein family is characterized by its diversity and abundance, which may due to a higher degree of compartmentalization of plant cells and a greater necessity for better water control ability [7]. For example, 35 aquaporin isoforms spread over all five chromosomes have been identified in *Arabidopsis thaliana* [8], 71 in cotton (*Gossypium hirsutum* L.) [9]; 41 in potato (*Solanum tuberosum*) [10], 55 in poplar (*Populus trichocarpa*) [11], and 45 in cassava (*Manihot esculenta* Crantz) [12]. Moreover, AQPs have versatile physiological roles in combatting abiotic stresses, which has been supported by analysis of transgenic plants with modified expression of various aquaporins, or from analysis of aquaporin mutants. For example, overexpression of *PeTIP4;1–1*, an aquaporin gene involved in bamboo shoot growth, confers drought and salinity tolerance in transgenic Arabidopsis [13]. Ectopically expressing apple *MdPIP1;3* increased fruit size and enhanced drought tolerance of transgenic tomatoes [14]. Overexpression of *ThPIP2;5* in transgenic *Tamarix* and *Arabidopsis* plants increases salt and osmotic stresses tolerance through improving ROS-scavenging capability, and reducing membrane damage compared to equivalent controls [15]. Taken together, mining the key AQPs genes controlling crop tolerance to salt becomes increasingly important for modern agriculture, especially via high throughput technologies.

Cucumber is one of the most commercially important vegetables worldwide and is sensitive to salt stress [16]. Although information on aquaporins of some plants has been well documented, very little is known about aquaporins in cucumber. Given the potential value of aquaporins in improving stress tolerance, it is necessary to identify aquaporin genes in cucumber. Thus, in this work, a genome-wide analyses of sequence, structural characteristics, chromosomal distribution, subcellular localization, exon-intron organization, conserved motifs, and expression patterns of putative CsAQPs were carried out. RNA-seq was used to determine the expression patterns of AQP genes which are likely involved in salt stress response. Furthermore, abiotic stress responses and water transport regulation function of several AQPs were investigated using qRT-PCR and water loss rate analysis. The knowledge obtained from this study is expected to provide a basis for exploring the functions and mechanisms of cucumber AQP proteins.

## Results

### Identification and classification of cucumber aquaporin genes

**Fig. 1:**
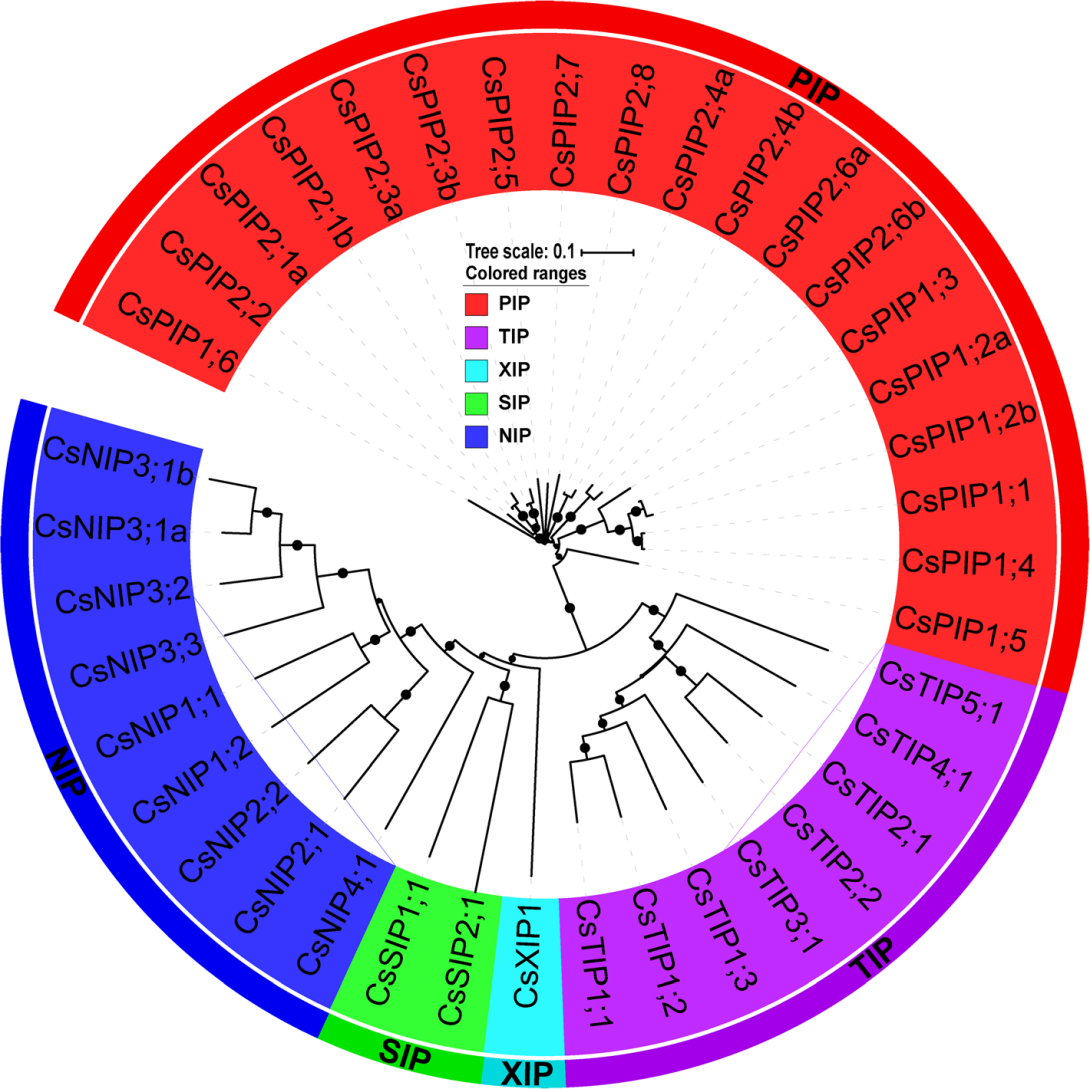
Phylogenetic analysis of 39 aquaporins identified in cucumber. Predicted amino acid sequences were aligned using ClustalW2 and the phylogenetic tree was constructed using MEGA7.0 software with the maximum likelihood method (1000 replicates). Different colors represent different aquaporin subfamilies

**Fig. 2:**
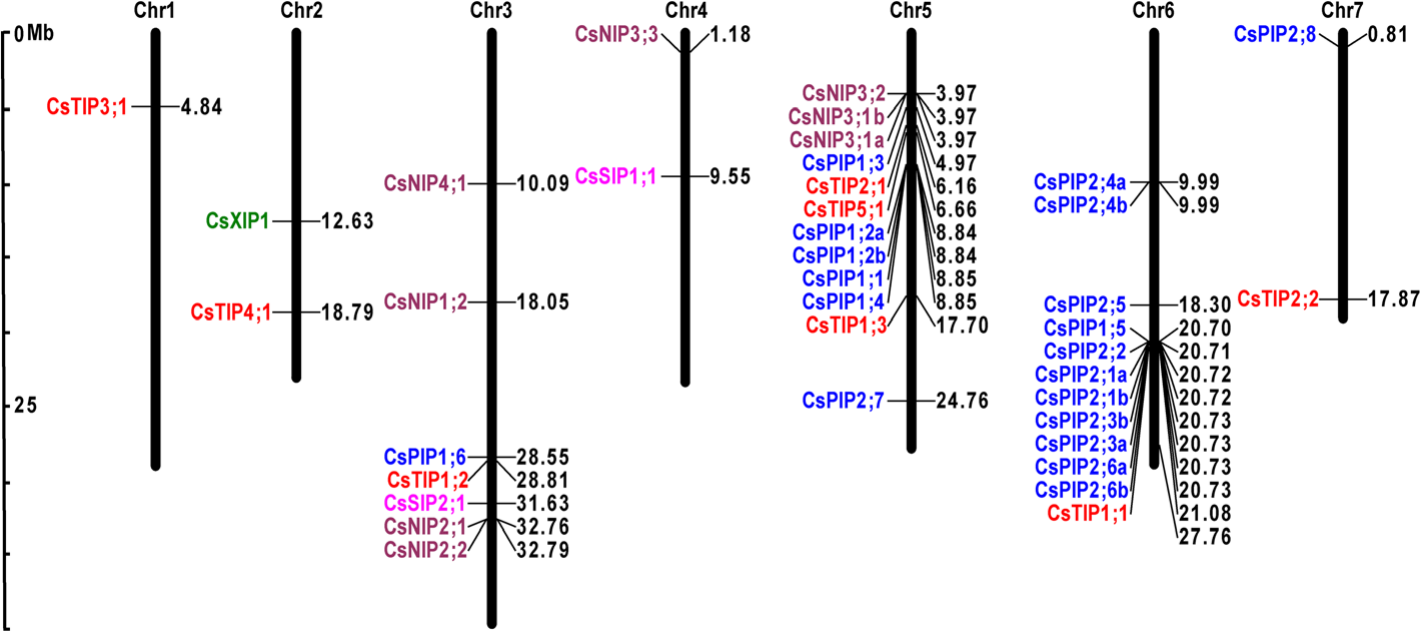
Distribution of the aquaporin genes on 7 cucumber chromosomes. Chr1–7 represent the chromosome 1 to 7. The rule on the left indicates the physical map distance among genes (Mbp). Blue, red, brown, pink, and green colors represent for PIPs, TIPs, NIPs, SIPs, and XIP, respectively

Sequence homology analysis and protein domain validation using Pfam led to the identification of 41 aquaporin-like genes in cucumber (Table 1). Among them, two genes (Csa6M445100, Csa7M336420) encoding partial aquaporin-like sequences, which are truncated and lacking any of the NPA motifs, were excluded from further sequence analysis. Consequently, in cucumber, 39 full-length protein-coding aquaporin genes were identified (Table 1). To systematically classify cucumber aquaporin genes and uncover the evolutionary relationship with aquaporin genes from other plants, an unrooted phylogenetic tree was constructed with MEGA7 using Neighbor-joining analysis (Additional file 2: Figure S1). Since no XIPs were detected in Arabidopsis, rice, maize, and potato, additional XIPs identified from *Ricinus communis*, *Hevea brasiliensis*, and *Hevea brasiliensis* were also used in phylogenetic analysis [17]. By comparing amino acid sequences of cucumber aquaporins with previously identified plant aquaporins, the 39 identified CsAQPs were divided as 19 CsPIPs (7 CsPIP1s and 12 CsPIP2s), 8 CsTIPs (3 CsTIP1s, 2 CsTIP1s, 1 CsTIP3, 1 CsTIP4, and 1 CsTIP5), 9 CsNIPs (2 CsNIP1s, 2 CsNIP2s, 4 CsNIP3s, and 1 CsNIP4), 2 CsSIPs (CsSIP1 and CsSIP2) and 1 CsXIP (Table 1; Fig. 1). Suffices (a, b) were used to denote splice variants derived from the same gene. Information including gene names, accession numbers, the length of deduced polypeptides, and protein structure features are presented in Table 1. We further mapped 39 CsAQP genes onto 7 chromosomes to identify their physical locations (Fig. 2). The 39 CsAQPs were located across all chromosomes, and chromosomal distribution of these genes varied greatly from one in chromosome 1, to a high of 13 in chromosome 6. Chromosomes 3, 5, 6, and 7 contained three to four subfamilies of aquaporin genes, whereas chromosomes 1, 2, and 4 carried one or two subfamilies of aquaporin genes (Fig. 2).

**Table 1:** Protein information, conserved amino acid residues, transmembrane domains (TMDs), and their predicated subcellular localization of *Cucumis sativus* aquaporins

Name	Gene ID	^a^Length	^b^MW	^c^pI	^d^GRAVY	^e^TM	Ar/R selectivity filter				NPA motifs		Froger’s positions					^f^Loc
							H2	H5	LE1	LE2	LB	LE	P1	P2	P3	P4	p5	
CsPIP1;1	Csa5M199270.1	292	31.43	7.67	0.31	6	F	H	T	R	NPA	NPA	Q	S	A	F	W	plas
CsPIP1;2a	Csa5M198770.1	292	31.37	7.68	0.34	6	F	H	T	R	NPA	NPA	Q	S	A	F	W	plas
CsPIP1;2b	Csa5M198770.2	217	23.20	7.62	0.21	4	F	/	/	/	NPA	/	Q	/	/	/	/	plas
CsPIP1;3	Csa5M153020.1	286	30.76	9.23	0.36	6	F	H	T	R	NPA	NPA	Q	S	A	F	W	plas
CsPIP1;4	Csa5M199280.1	292	31.40	7.67	0.29	5	F	H	T	R	NPA	NPA	Q	S	A	F	W	plas
CsPIP1;5	Csa6M445090.1	191	20.26	9.51	0.47	4	/	N	A	R	NPA	NPA	K	S	A	F	W	plas
CsPIP1;6	Csa3M739030.1	290	31.25	9.10	0.49	6	F	H	T	R	NPA	NPA	Q	S	A	F	W	plas
CsPIP2;1a	Csa6M445130.1	284	29.92	7.04	0.51	6	F	H	T	R	NPA	NPA	Q	S	A	F	W	plas
CsPIP2;1b	Csa6M445130.2	208	21.92	6.18	0.45	4	F	S	/	/	NPA	/	Q	/	/	/	/	plas
CsPIP2;2	Csa6M445120.1	284	30.24	8.78	0.56	6	F	H	T	R	NPA	NPA	Q	S	A	F	W	plas
CsPIP2;3a	Csa6M445140.1	284	29.98	7.64	0.56	7	F	A	F	T	NPA	HLA	Q	I	G	F	N	plas
CsPIP2;3b	Csa6M445140.2	208	21.93	6.26	0.56	4	F	S	/	/	NPA	/	Q	/	/	/	/	plas
CsPIP2;4a	Csa6M140850.1	283	30.24	7.63	0.47	6	F	H	T	R	NPA	NPA	Q	S	A	F	W	plas
CsPIP2;4b	Csa6M140850.2	252	26.85	6.06	0.47	5	F	H	T	R	NPA	NPA	Q	S	A	S	L	plas
CsPIP2;5	Csa6M405320.1	276	29.37	9.36	0.53	6	F	H	T	R	NPA	NPA	Q	S	A	F	W	plas
CsPIP2;6a	Csa6M445150.1	279	29.72	8.97	0.62	6	F	H	T	R	NPA	NPA	Q	S	A	F	W	plas
CsPIP2;6b	Csa6M445150.2	197	20.96	9.75	0.69	5	F	H	T	R	NPA	NPA	Q	S	A	F	W	plas
CsPIP2;7	Csa5M623360.1	287	30.52	9.11	0.41	6	F	H	T	R	NPA	NPA	Q	S	A	F	W	plas
CsPIP2;8	Csa7M014450.1	280	29.85	9.24	0.45	6	F	H	T	R	NPA	NPA	M	S	A	F	W	plas
CsTIP1;1	Csa6M448110.1	250	25.72	5.64	0.78	7	H	I	A	V	NPA	NPA	F	A	S	Y	W	vacu
CsTIP1;2	Csa3M743400.1	253	26.29	6.03	0.93	7	H	I	A	V	NPA	NPA	F	A	A	Y	W	vacu
CsTIP1;3	Csa5M505790.1	254	26.53	5.30	0.71	6	H	I	A	V	NPA	NPA	F	T	A	Y	W	vacu
CsTIP2;1	Csa5M162580.1	250	25.09	5.39	0.99	6	H	I	G	R	NPA	NPA	F	S	A	Y	W	plas
CsTIP2;2	Csa7M447100.1	248	25.44	5.66	0.87	6	H	I	G	R	NPA	NPA	Y	S	A	Y	W	vacu
CsTIP3;1	Csa1M043290.1	289	30.66	7.17	0.46	5	H	I	A	R	NPA	NPA	L	A	S	Y	W	vacu
CsTIP4;1	Csa2M374630.1	247	25.72	6.01	0.90	7	H	I	A	R	NPA	NPA	Y	S	A	Y	W	vacu
CsTIP5;1	Csa5M168860.1	255	26.12	6.88	0.86	6	N	V	G	C	NPA	NPA	A	A	A	Y	W	plas
CsNIP1;1	Csa6M520340.1	276	29.45	9.58	0.54	6	W	V	A	R	NPA	NPA	F	S	A	Y	I	plas
CsNIP1;2	Csa3M345890.1	269	28.88	7.67	0.78	5	W	V	A	R	NPA	NPA	F	S	A	Y	M	vacu
CsNIP2;1	Csa3M826640.1	288	30.55	9.40	0.33	6	G	S	G	R	NPA	NPV	L	T	A	Y	F	vacu/plas
CsNIP2;2	Csa3M826650.1	261	27.62	6.05	0.45	6	C	S	G	R	NPA	NPA	M	S	A	Y	M	plas
CsNIP3;1a	Csa5M146200.1	249	26.01	8.86	0.65	4	A	I	G	R	NPS	NPV	F	T	A	Y	M	plas
CsNIP3;1b	Csa5M146200.2	221	23.51	8.50	0.74	6	A	I	/	/	NPS	/	F	/	/	F	l	plas
CsNIP3;2	Csa5M146190.1	298	30.80	8.63	0.50	5	A	I	G	R	NPS	NPV	F	A	A	Y	l	plas
CsNIP3;3	Csa4M007030.1	304	31.52	8.26	0.43	5	T	V	A	R	NPA	NPV	F	T	A	Y	l	plas
CsNIP4;1	Csa3M149960.1	268	28.45	6.88	0.68	6	A	V	A	R	NPA	NPA	F	S	A	Y	I	plas/vacu
CsSIP1;1	Csa4M192210.1	243	25.59	9.41	0.83	6	V	T	P	N	NPT	NPA	F	A	A	Y	W	vacu
CsSIP2;1	Csa3M816140.1	238	25.93	10.00	0.66	5	F	K	G	S	NPL	NPA	F	V	A	Y	W	vacu
CsXIP1;1	Csa2M263850.1	319	34.47	8.27	0.66	7	V	I	V	R	SPI	SPA	Y	C	A	F	W	cyto
/	Csa6M445100.1	106	11.57	4.51	0.62	2	F	/	/	/	/	/	/	/	/	/	/	plas
/	Csa7M336420.1	99	10.66	4.83	0.72	3	/	/	/	/	/	/	/	/	/	/	/	mito

^a^*Length* Protein length (aa)

^b^*MW* Protein molecular weight (kD)

^c^*pI* isoelectric point

^d^*GRAVY* Grand average of hydrophobicity

^e^TM represents for the numbers of Transmembrane helices predicted by TMHMM Server v.2.0 tool

^f^Best possible cell localization predicted by the WoLF PSORT tool (*Chlo* Chloroplast, *Cyto* Cytosol, *Plas* Plasma membrane, *Vacu* Vacuolar membrane)

*LB* Loop B, *LE* Loop E. *NPA* Asparagine-Proline-Alanine, *H2* represents for Helix 2, *H5* represents for Helix 5, *LE1* represents for Loop E1, *LE2* represents for Loop E2, *Ar/R* represents for Aromatic/Arginine

### Features of aquaporin proteins

**Fig. 3:**
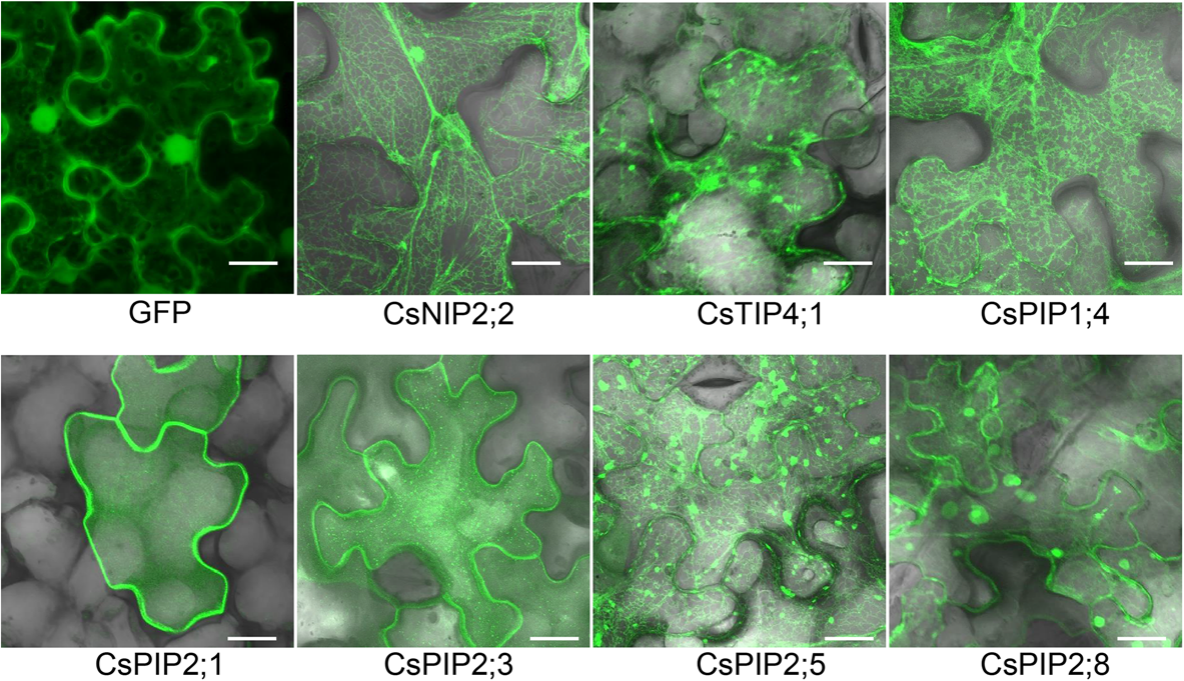
Subcellular localization of CsAQPs. The free GFP (positive control), as well as the AQP:GFP fusion proteins of CsNIP2;2, CsTIP4;1, CsPIP1;1, CsPIP2;1, CsPIP2;3, CsPIP2;5, and CsPIP2;8, were transiently expressed in tobacco leaves via *Agrobacterium tumefaciens* strain GV3101. Subcellular localization was then observed by confocal laser scanning microscopy after 48 h from the infiltration. Scale bar = 20 μm

Sequence analysis showed that the 39 deduced CsAQP proteins ranged from a minimum of 191 to a maximum of 319 amino acids, with predicted sizes ranging from 20.96 to 34.47 kDa, and the isoelectric point (pI) values ranging from 4.51 to 10.00 (Table 1). Positive and negative scores for protein grand average hydrophobicity (GRAVY) reflected hydrophobicity and hydrophilicity, respectively. The GRAVY results were all positive, ranging from 0.205 to 0.989, which indicated that they were all hydrophobic proteins, which is a necessary characteristic for AQPs (Table 1). Sub-cellular localizations of CsAQPs were predicted to ascertain expression at different cellular/organellar levels (Table 1). Based on subcellular localization prediction, all CsPIPs were likely localized to plasma membrane (Table 1). Most CsTIPs (6) were predicted to localize in vacuoles while 2 of them were predicted to localize in plasma membranes. Large majority of CsNIPs were predicted to localize in plasma membranes, while CsSIPs were localized to vacuoles and CsXIP localized to cytoplasm (Table 1). To further confirm the predication, seven CsAQPs were fusion to green fluorescent protein and were transient expressed in *Nicotiana benthamiana* leaves using *Agrobacterium tumefaciens*-mediated approach. Overall, consistent with the predication, the transient expression analyses provide evidence that CsAQPs were mostly membrane or endomembrane localized. CsNIP2;2 and CsPIP1;4 were mainly localized to endoplasmic reticulum (ER); CsPIP2;1 and CsPIP2;3 were mainly localized to plasma membrane; CsTIP4;1, CsPIP2;5, and CsPIP2;8 were localized to multiple positions, including ER, plasma membrane, and endomembrane (Fig. 3). The diverse and broad array of sub-cellular localizations of plant AQPs reflects the high degree of compartmentation of plant cells and the need for cells to control water and/or solutes transport across the plasma membrane as well as across intracellular membranes.

### Motif composition and gene structure analyses

**Fig. 4:**
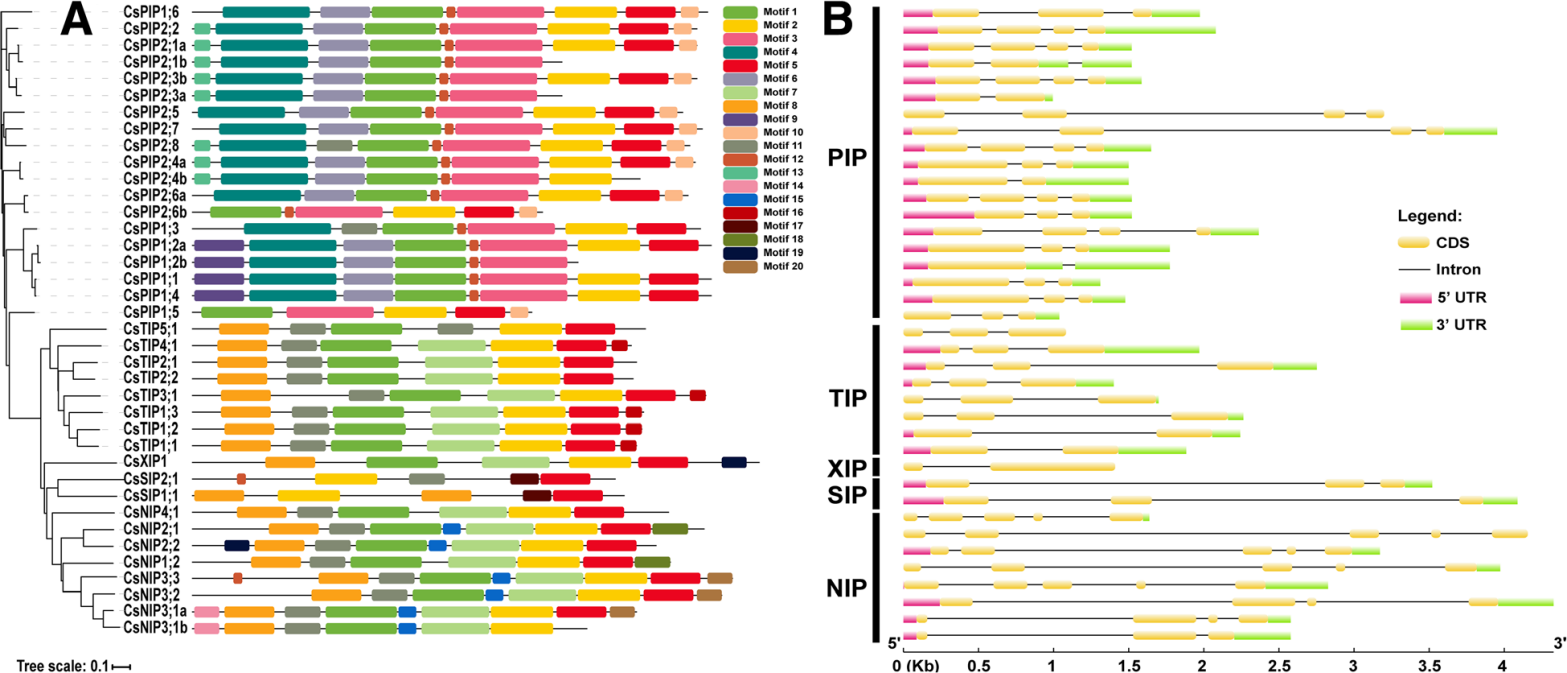
Conserved motifs of aquaporin proteins and exon-intron organization of corresponding coding genes in cucumber. **a** The motifs were identified by MEME tools. Twenty motifs (1–20) were indicated by different colored numbers (1–20). **b** Exon-Intron structure of 39 cucumber aquaporins genes. Untranslated Regions (UTRs) are represented by red and green boxes, coding regions (CDS) are shown as yellow round-corner rectangle, and introns are shown as black lines

The protein motifs are highly conserved amino acid residues that are considered to possibly have functional and/or structural roles in active proteins [18]. In this study, motif distributions of 39 CsAQP proteins were analyzed using the MEME program, and 20 conserved motifs, designated as motif 1 to motif 20, were identified (Fig. 4a). Most CsAQP proteins of the same subfamily generally had similar motifs. Of these, motif 1 was found in all the CsAQPs, except for CsSIP1;1. The protein sequences of PIP subfamily members shared high similarity. Motifs 1 and 3 were commonly detected in PIP subfamily members. Some clusters contain several relatively specific motifs. For example, motifs 10 and 13 were found only in subfamily CsPIP, while motif 20 was found only in subfamily CsNIP. In most cases, splice variants of CsAQPs showed similar protein sequences with a loss of first or last 2–3 motifs.

Most cucumber aquaporin sequences showed a typical topology of six transmembrane helical domains (TMs); PIP1;2, PIP1;5, PIP2;1b, PIP2;3b, and NIP3;1a showed four TMs; PIP1;4, PIP2;4a, PIP2;6a, TIP 3;1, NIP1;2, NIP3;2, NIP3;3, and SIP2;1 showed five TMs, whereas PIP2;3a, TIP1;1, TIP1;2, TIP4;1, and XIP1 showed seven TMs (Table 1). Sequence similarity of PIP members varied between 26.67 (CsPIP1;5 and CsPIP1;2b) and 99.66% (CsPIP1;4 and CsPIP1;1), whereas 27.57–80.69% sequence similarity was observed between CsPIP1 and CsPIP2 members. CsTIP and CsNIP subfamily showed 31.38–84.58% and 40.63–77.11% similarity within the subfamily respectively (Additional file 1: Table S4). Within CsTIP1 and CsTIP2 members, 34.45–84.58% and 34.57–81.60% amino acid sequence similarity were observed, respectively. CsSIP1;1 and CsSIP2;1 shared 48.57% sequence similarity.

Exon-intron structural diversity often plays a key role in the evolution of gene families and can provide additional evidence to support phylogenetic groupings [19]. To seek further insights into gene structure, the intron-exon structures of the cucumber AQPs were analyzed. The number of introns in CsAQPs ranged from one to four. Most members (17) had two introns, while 10 members had three introns, and six members had 4 or 1 introns (Fig. 4b, Additional file 1: Table S1). The fewest number of introns were observed in *CsXIP1;1*, *CsTIP1;1*, and *CsPIP 2;3b*. Several genes, including *CsPIP2;5*, *CsTIP5;1*, *CsXIP1*, and *CsNIP2;1*, did not have UTRs at both 5′ and 3′ ends. CsSIPs, two CsPIPs (*CsPIP2;5*, *CsPIP2;7*), and most members of CsNIP had long introns ranging from 77 bp (*CsNIP4;1*) to 2337 bp (*CsNIP2;1*). Overall, cucumber aquaporins showed a complex gene structure with varying intron positions and lengths.

#### Asn-Pro-Ala (NPA) motifs

Usually, two highly conserved Asn-Pro-Ala (NPA) motifs that create an electrostatic repulsion of protons and form the water pore, and the aromatic/Arg (ar/R) selectivity filter are essential for selective transport of substrate molecules [20]. Point mutations of the amino acid at these positions have been found to strongly affect the substrate specificities of aquaporins [21]. To understand the possible physiological role and substrate specificity of cucumber aquaporins, NPA motifs, residues at ar/R selectivity filter (H2, H5, LE1 and LE2), and Froger’s positions (P1 to P5) were identified and analyzed (Table 1). As can be seen in Table 1, most CsPIPs and all CsTIPs harbored 2 conserved NPA motifs in LB and LE, except for CsPIP1;2, CsPIP2;1b, CsPIP2;3a, and CsPIP2;3b, which were found to contain single NPA motif. In CsPIP2;3a, the second NPA motif was replaced by a HLA motif. CsSIP aquaporins showed a conserved second NPA motif, but all of the first NPA motifs showed a replacement of Alanine (A) by Threonine (T) (CsSIP1;1) or Leucine (L) (CsSIP2;1). In the CsNIP sub-family, the first NPA motif showed an Alanine (A) to Serine (S) substitution in three CsNIPs (CsNIP3;1a, CsNIP3;1b, and CsNIP3;2), and the second NPA motif showed an Alanine (A) to Valine (V) substitution in four CsNIPs (CsNIP2;1, CsNIP3;1a, CsNIP3;2, and CsNIP3;3). In the CsXIP sub-family, CsXIP1 encoded dissimilar first (Serine, S) and third (Isoleucine, I; Alanine, A) amino acids in both NPA motifs.

#### Aromatic/Arginine (ar/R) selectivity filter and Froger’s positions

Compared to the two NPA motifs, the ar/R positions showed an increased family-specific sequence. CsPIPs could represent an ancestor aquaporins that have been conserved throughout evolution of terrestrial plants [22]. Most CsPIP members contained highly conserved amino acids in the ar/R selectivity filter (F-H-T-R) with the exception of CsPIP2;3a, CsPIP2;3b, CsPIP1;5, CsPIP1;2, and CsPIP2;1b. As to Froger’s positions, 13 out of 19 CsPIP members exhibited identical amino acids in the Froger’s positions (Serine-Alanine-Phenylalanine-Tryptophan, S-A-F-W) except for CsPIP2;3a, CsPIP1;5, CsPIP2;8, and CsPIP2;4b. CsPIP2;3b, CsPIP2;1b, and CsPIP1;2b lacked the P3-P5 position as they did not contain the last transmembrane domains.

In the CsTIPs, P3, P4, and P5 positions were highly conserved, but the selectivity filter (ar/R) was highly variable across plant species including potato, flax, and *Jatropha curcas* [10, 17, 23]. Similar results were also found in CsTIPs. The P1 position of CsTIPs was highly variable, as four amino acids, Phenylalanine (F), Tyrosine (Y), Leucine (L), or Alanine (A), alternatively appeared in this position. P2-P3 positions are also variable with 2 to 3 alternative amino acids. In addition, P4-P5 were highly conserved, which exhibited no variation as expected Tyrosine (Y) and Tryptophan (W) amino acids respectively were identified. In the CsTIP sub-family, the ar/R is formed by Histidine/Asparticacid (H/A) in H2; Isoleucine/Valine (I/V) in H5, Alanine/Glycine (A/G) in LE1, and Valine/Arginine/Cysteine (V/A/C) in LE2. P4 and P5 positions were highly conserved with a Tyrosine (Y) and Tryptophan (W), respectively.

CsNIPs, CsSIP, and CsXIP showed a highly variable ar/R selectivity filter and Froger’s positions, with the exception of LE2 and P3 in CsNIPs. The CsXIP sub-family member showed Valine/Histidine (V/H, H2), Isoleucine (I, H5), Valine/Glycine (V/G, LE1), Arginine (R, LE2) of ar/R filter residues, and Tyrosine (Y, P1), Cysteine/serine (C/S, P2), Alanine (A, P3), Phenylalanine/Tyrosine (F/Y, P4), Tryptophan (W, P5) in the Froger’s positions.

### *Cis*-acting regulatory elements analysis

**Fig. 5:**
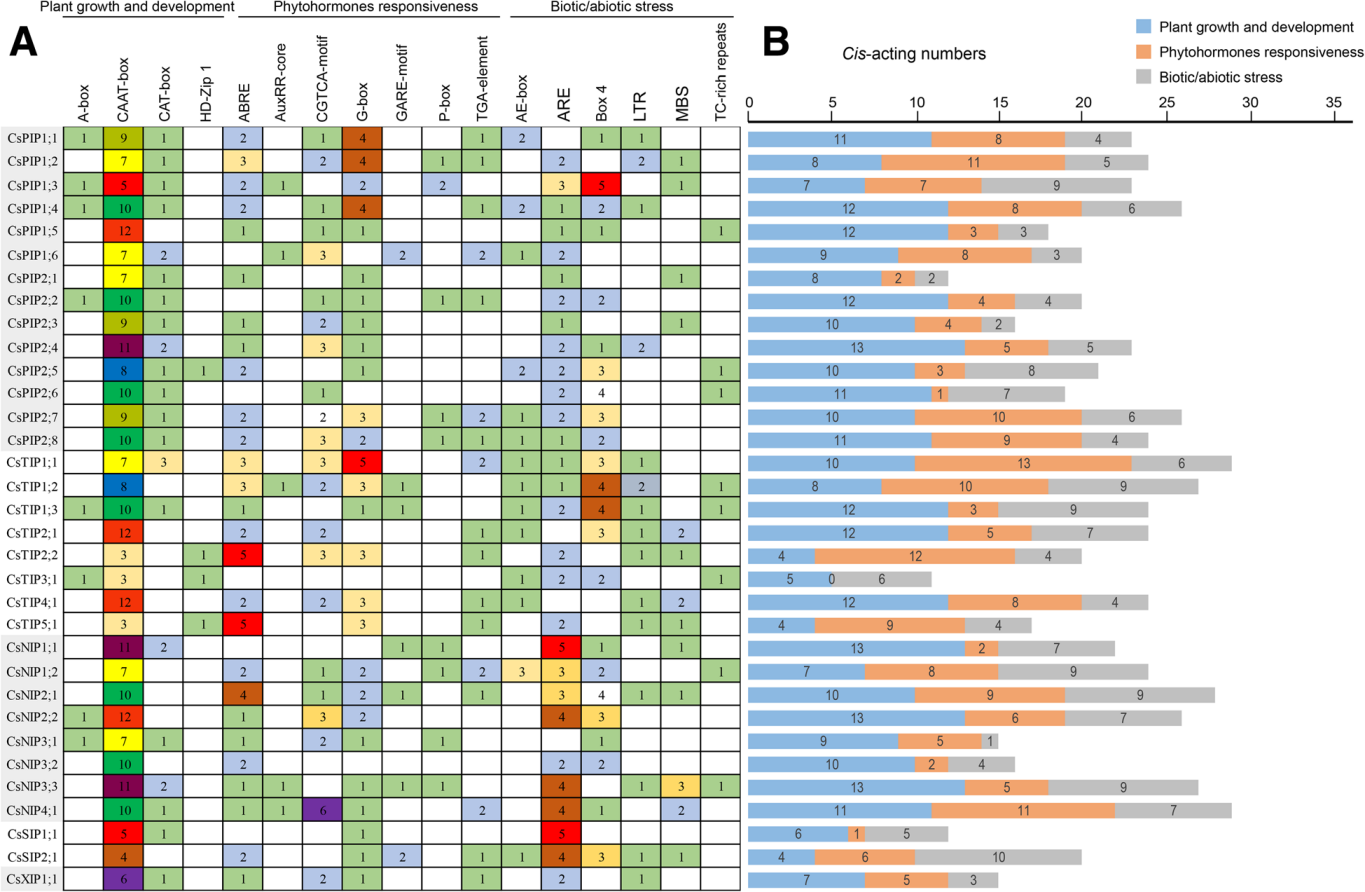
Analysis of *cis*-acting element numbers in cucumber aquaporin genes. **a** The different colors and numbers of the grid indicated the numbers of different promoter elements in these aquaporin genes. **b** The different colored histogram represented the sum of the *cis*-acting elements in each category

The *cis*-acting regulatory element was a specific motif that binds to an appropriate transcription factor to regulate gene transcription in plants [24]. To identify putative *cis*-acting elements in the promoter region, we scanned the 2000 bp upstream promoter regions of AQP genes before transcriptional start site (ATG). A total of 721 *cis*-acting elements were observed in the promoter regions of AQP family genes (Additional file 1: Table S5). Stress-related cis-regulatory elements that were identified in the cucumber AQPs included low-temperature responsive element (LTR), light responsive element (G-box), salicylic acid responsive element (TCA-element), anaerobic induction regulatory element (ARE), defense and stress responsive element (TC-rich repeats), ABA responsive element (ABRE), MYB binding site involved in drought-inducibility (MBS), auxin-responsive element (TGA-element), MeJA-responsive element (CGTCA-motif) (Fig. 5). These results further indicated that CsAQPs might participate in abiotic stress pathways. ARE motif that is essential for the anaerobic induction was present in all the AQP promoters except for *CsPIP1;1*, *CsPIP2;8*, and *CsTIP4;1*. Most AQP genes contain regulatory elements CGTCA-motif (30, *cis*-acting regulatory element involved in the MeJA-responsiveness), G-Box (28, *cis*-acting regulatory element involved in light responsiveness), ARBE (26, *cis*-acting element involved in the abscisic acid responsiveness) and ARE (31, *cis*-acting regulatory element essential for the anaerobic induction).

Various type of *cis*-acting elements and differences in transcription factors that bind to *cis*-regulatory elements in the AQP genes may be responsible for differential expression of aquaporin genes in response to environment stresses in the plant. Abscisic acid (ABA) plays a vital role in plant growth and development as well as in in mediating plant response to a wide range of stresses. The effect of ABA on AQP gene expression has been described for various plant species, which suggested that AQP gene expression was controlled in either an ABA-dependent or ABA-independent manner. ARBE is bound with ABA-responsive element binding factors (AREB) for ABA dependent pathway induction and were identified in most of the CsAQP genes (26). Moreover, it should be noted that other *cis*-elements involved in osmotic stress, such as MBS and TC-rich repeats, were also observed in CsAQPs promoters. This result suggested that these aquaporin members in cucumber may be regulated by various factors, including drought and ABA, which need to be experimentally proved in further studies.

### Homology modelling of aquaporin genes in cucumber

**Fig. 6:**
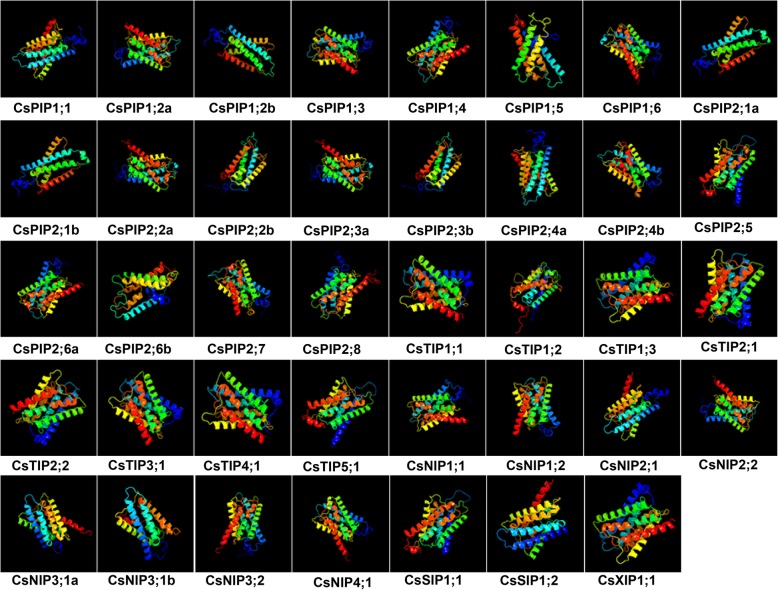
Predicted 3D models of cucumber aquaporin proteins. Models were generated by using Phyre2 server. Models were visualized by rainbow color from N to C terminus. Two templates, c2w2eA and d1j4na from structures of Yeast aquaporin were used in the modelling of CsPIP sub-family. Among them, template d1j4na were used in CsPIP1;5, CsPIP2;5, and CsPIP2;6b modelling; the rest of the members were modelling with template c2w2eA (from Yeast). In the CsTIP sub-family, template c5i32A (from *Arabidopsis thaliana*) were used in CsTIP1;1, CsTIP1;3, CsTIP2;1; CsTIP2;2, CsTIP3;1, and CsTIP4;1 modelling; template c2w2eA (from Yeast) was used in CsTIP1;2 modelling; template d1j4na was used in CsTIP5;1 modelling. Template c2w2eA (from Yeast) were used in CsNIP sub-family members modelling. Template d1j4na was used in CsSIP1;1 modelling; template c2w2eA (from Yeast) was used in CsSIP2;1 modelling. And template c5i32A (from *Arabidopsis thaliana*) was used in CsXIP1;1 modelling

All 39 cucumber aquaporin family members were three-dimension modelled using Phyre2 server (Fig. 6). Predicted models were based on the reported templates to heuristically maximize the alignment coverage, percentage identity, and confidence score for the tested sequences. In CsAQP proteins, the mainly predicted secondary structure was α-helix (57–76% in each CsAQP), whereas β-strands were only detected in CsPIPs (2–3% in CsPIP2;1a/b and CsPIP2;4b). Transmembrane (TM) helices was the most detected α-helix types and occupied for 46–59%. In addition, in order to clarify the similarity or difference of the generated models, the superposition structures were used to calculate the percentage of structural coverage. About 81–94% structural coverage were demonstrated between cucumber aquaporin proteins and corresponding model sequences, suggesting that the cucumber aquaporins structure prediction are highly reliable. 3D modeling results revealed that these aquaporins showed tertiary structures similarity, implying that cucumber aquaporins may evolved from same ancestor sequence and/or under purification selection force to keep stabilization during long-term acclimation after the initially divergent.

### Expression of AQP genes in cucumber

**Fig. 7:**
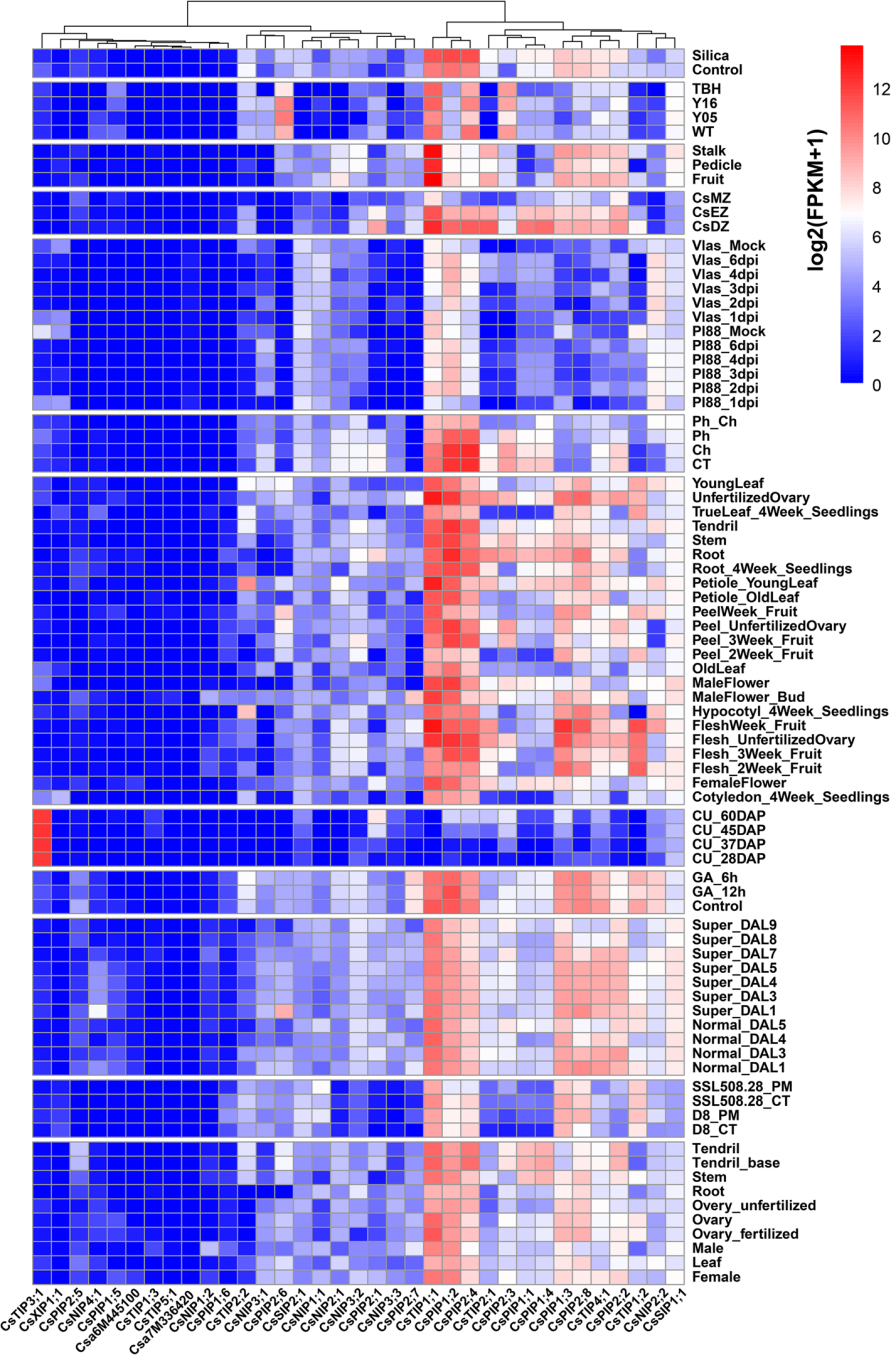
Expression pattern profiling of cucumber AQP genes. The different tissues used for expression analysis including root, leaf, seed, ovary, flower, fruit, pedicle, and stem. Experimental design: CT, control; DAP, day after pollination; DAL, the normal ovary blooms at 4–5 days after labeling (when the ovary is visible); tbh, a tiny branched hair mutant; Y05, fruit spine on fruits of 0.5 cm long; Y16, fruit spine on fruits of 1.6 cm long; WT, wild type cucumber; CsDZ, Root differentiation zone; CsEZ, Root elongation zone; CsMZ, Root meristematic zone; Ch, treated with chrysophanol; Ph, treated with physcion; GA_12h, treated with GA at 12 h; GA_6h, treated with GA at 6 h. Red indicates high concentrations, whereas low relative concentrations are deep blue. Data were normalized. Expression values are given after logarithmic transformation of RPKM (reads per kilobase of exon model per million mapped reads). All detailed information can be found in Cucurbit Genomics Database (http://cucurbitgenomics.org/organism/2)

RNA transcript profiling is an important strategy to study the expression of a large number of genes. Cucumber Illumina RNA-seq data were obtained from the Cucurbit Genomics Database (http://cucurbitgenomics.org/organism/2). The expression levels represented by FPKM values could be assigned to CsAQP genes. A heat map was created to characterize the expression patterns of CsAQP genes in different organs and in response to different treatments (Fig. 7; Additional file 1: Table S6). FPKM values were used to measure the transcription levels of the AQPs. According the heat map, many CsPIPs (e.g. *CsPIP1;2*, *CsPIP2;4*, *CsPIP1;3*, and *CsPIP2;8*) showed high expression levels and were expressed in all analyzed tissues, suggesting a possible role in constitutive transport processes of AQPs throughout the plant (Fig. 7; Additional file 2: Figure S2). Most CsTIPs transcripts were abundantly expressed in fruit and ovary compared to other tissues. For example, *CsTIP1;1*, *CsTIP1;2*, *CsTIP2;1*, *CsTIP2;4*, and *CsTIP4;1* showed relatively high abundance in almost every tissue, whereas downy mildew infection decreased its expression level to some extent, suggesting that stress-related biological functions of CsAQPs, which need to be further studied. In contrast, some CsTIPs, such as *CsTIP3;1*, *CsTIP1;3*, and *CsTIP5;1* were generally expressed at lower levels across different tissues as well as in response to different treatments (e.g. powdery mildew, silicon), except *CsTIP3;1*, which showed relatively higher expression level in the seed (Fig. 7; Additional file 2: Figure S2). All CsNIPs and CsXIP were moderately or lowly expressed. *CsNIPs1;1* was not expressed in any organ except very low expression level in ovary and in response to downy mildew infection. Several CsAQPs like *CsTIP1;1*, *CsTIP2;1*, and *CsPIP1;3* were found to be expressed in dynamic, fruit-specific patterns, indicating a role in transport of water or solutes during fruit development as suggested in tomato [25]. Heterotetramerization of plant aquaporins, especially between PIP1 and PIP2 isoforms, have been previously reported [26]. To identify possible interacting proteins, we performed a co-expression analysis among the different cucumber organs (e.g. leaf, ovary, floral, root, fruit) and under different growth environment (e.g. silicon, physcion treatment) (Additional file 2: Figure S3). We found three pairs of *CsPIPs* (*CsPIP1;3* and *CsPIP2;8*, *CsPIP1;4* and *CsPIP1;1*, *CsPIP2;4* and *CsPIP1;2*) with a Pearson correlation coefficient > 0.8, suggesting their possible functional interaction, which needs further experimental confirmation.

### Effect of salt stress on plant growth, H_2_O_2_ content and plant hydraulic properties

**Fig. 8:**
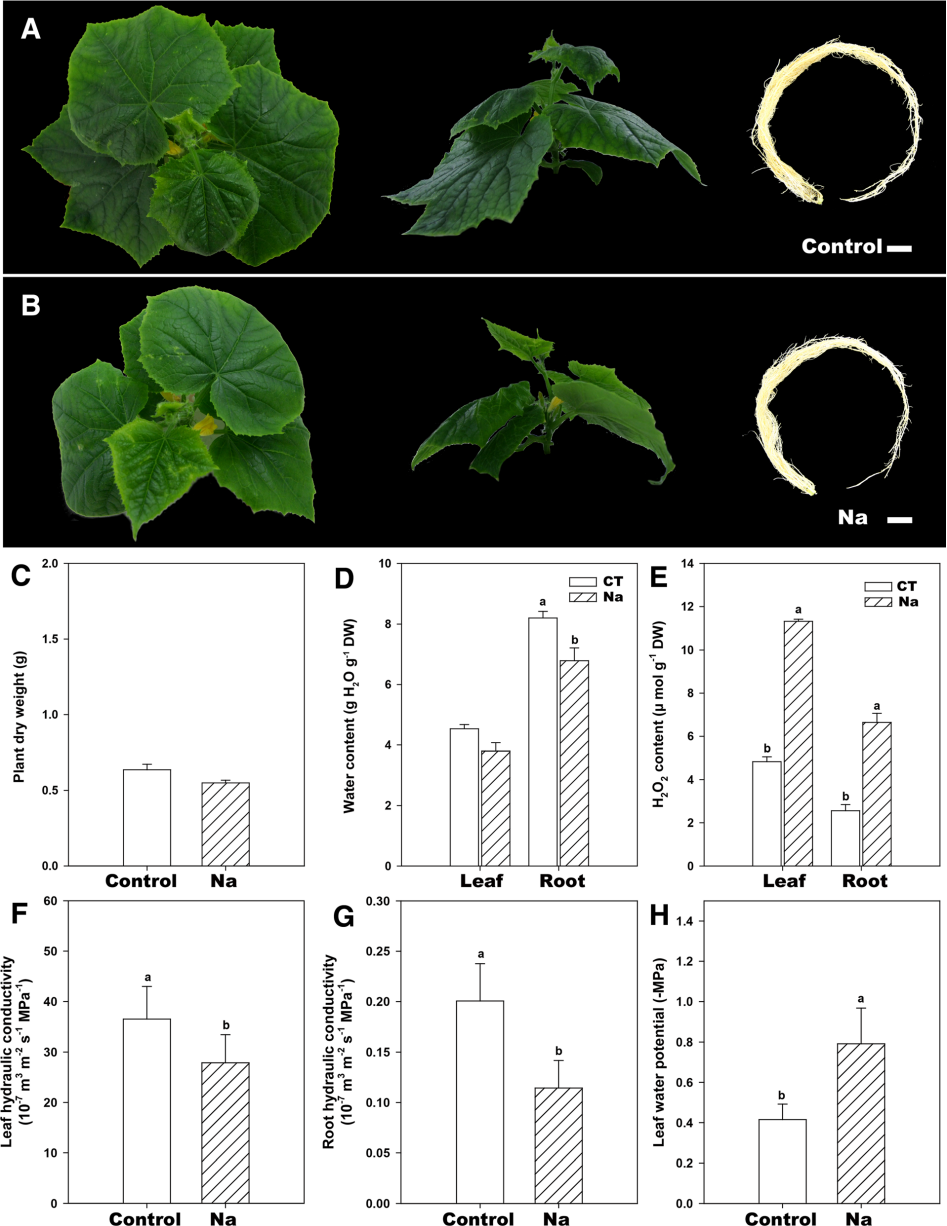
Effect of salt stress in cucumber. **a**, **b** The symptoms of salt injury in cucumber exposed to 3 days salt stress. Effect of salt on (**c**) plant total dry weight, **d** water content, **e** H_2_O_2_ content, **f**, **g** plant hydraulic conductance and (**h**) leaf water potential of cucumber seedlings. These parameters were determined after 3 d of salt treatment. Data are means ± SD (*n* = 8 for plant total dry weight, total water content, 5 for H_2_O_2_ content, plant hydraulic conductance, and leaf water potential). Different letters in a column indicate significant differences between the treatments at *p* < 0.05 level

As shown in Fig. 8c, d, salinity slightly decreased plant total dry weight and leaf total water content, and significantly decreased root total water content. Furthermore, salt stress usually induces ROS accumulation in plants, causing oxidative stress [27]. Compared with control, salt stress significantly increased H_2_O_2_ contents in both leaves and roots (Fig. 8e). Root hydraulic conductance can be used to assess root water uptake rate while leaf-specific conductivity reflects the capacity of stem to supply leaves with water [28]. In this study, cucumber leaf and root hydraulic conductance were significantly decreased after 3 days of salt stress (Fig. 8f, g), which is consistent with the decreased water content. The early response of many plants to salinity include the reduction of leaf water potential [29]. Similarly, as can been seen in Fig. 8h, we also found cucumber leaf water potentials were significantly decreased by 75 mM NaCl treatment.

### Transcriptomic profiling of the salt-stress response of AQPs in cucumber

**Fig. 9:**
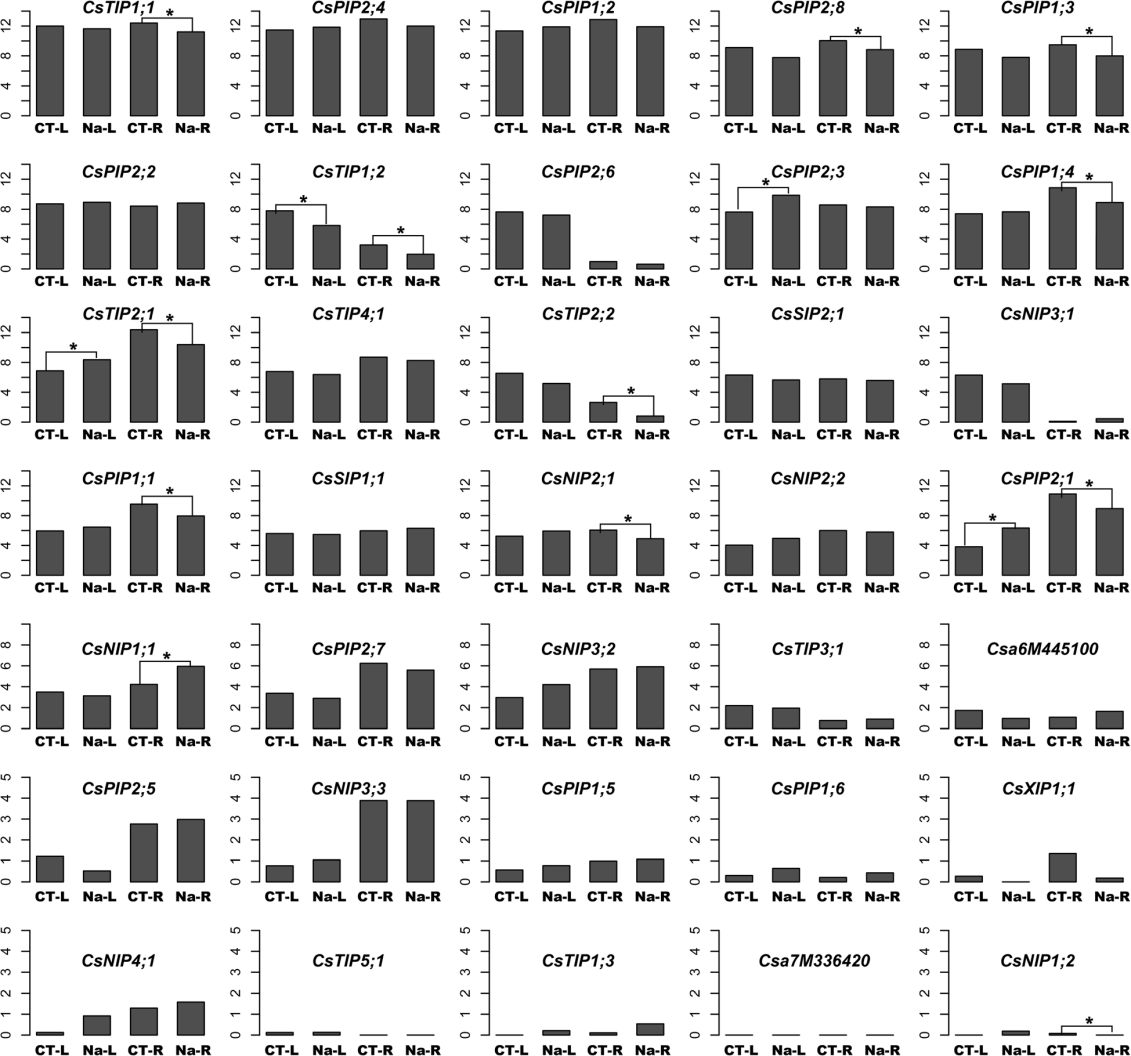
Regulating patterns of cucumber AQP genes in responding to salt stress. CK-L and Na-L, control and salt stressed samples of leaves; CK-R and Na-R, control and salt stressed samples of roots. Y-axis represents the expression levels of corresponding genes which indicates as log2(FPKM+1). * represents significantly differentially expressed genes (fold change ≥2, *p* value and FDR ≤ 0.05)

Salinity is a major environmental factor limiting productivity and distribution of plants. Previous research has shown that salt stress response of aquaporins is highly variable depending on aquaporin isoform, stress levels, tissue, and species [30]. In order to understand the response pattern of cucumber AQPs to salt stress, roots collected from CK and NaCl-treated seedlings were used for RNA-sequencing analysis. More than 139 million reads were generated from CK and Na samples (three biological replicates for each sample were performed). As a result, a total of 20,079 (86.37%) expressing genes were assembled (Additional file 1: Table S7). And consistent with RNA-seq data obtained from NCBI, RNA-seq data generated in our research suggested that *CsPIP1–2*, *CsPIP1–3*, *CsPIP2–4*, *CsPIP2–8*, and *CsTIP1–1* were among the most abundant aquaporins in the root of cucumber, accounting for 80% of all CsAQP transcripts (Fig. 9, Additional file 1: Table S8). Moreover, expression levels of these AQPs were significantly decreased by salt stress, which may account for the NaCl-induced down-regulation of hydraulic conductance. In the leaves, according to our RNA-seq data released previously (GSE116265) [31], CsAQPs showed varied expression patterns in response to salt stress. Among the top ten abundantly expressed AQPs in leaf tissues, the number of AQP genes induced by salt stress (*CsPIP2;4*, *CsPIP1;2*, *CsPIP2;2*, *CsPIP2;3*, and *CsPIP1;4*) were equal to that of repressed ones (*CsTIP1;1*, *CsPIP2;8*, *CsPIP1;3*, *CsTIP1;2*, *CsPIP2;6*).

### qRT-PCR

**Fig. 10:**
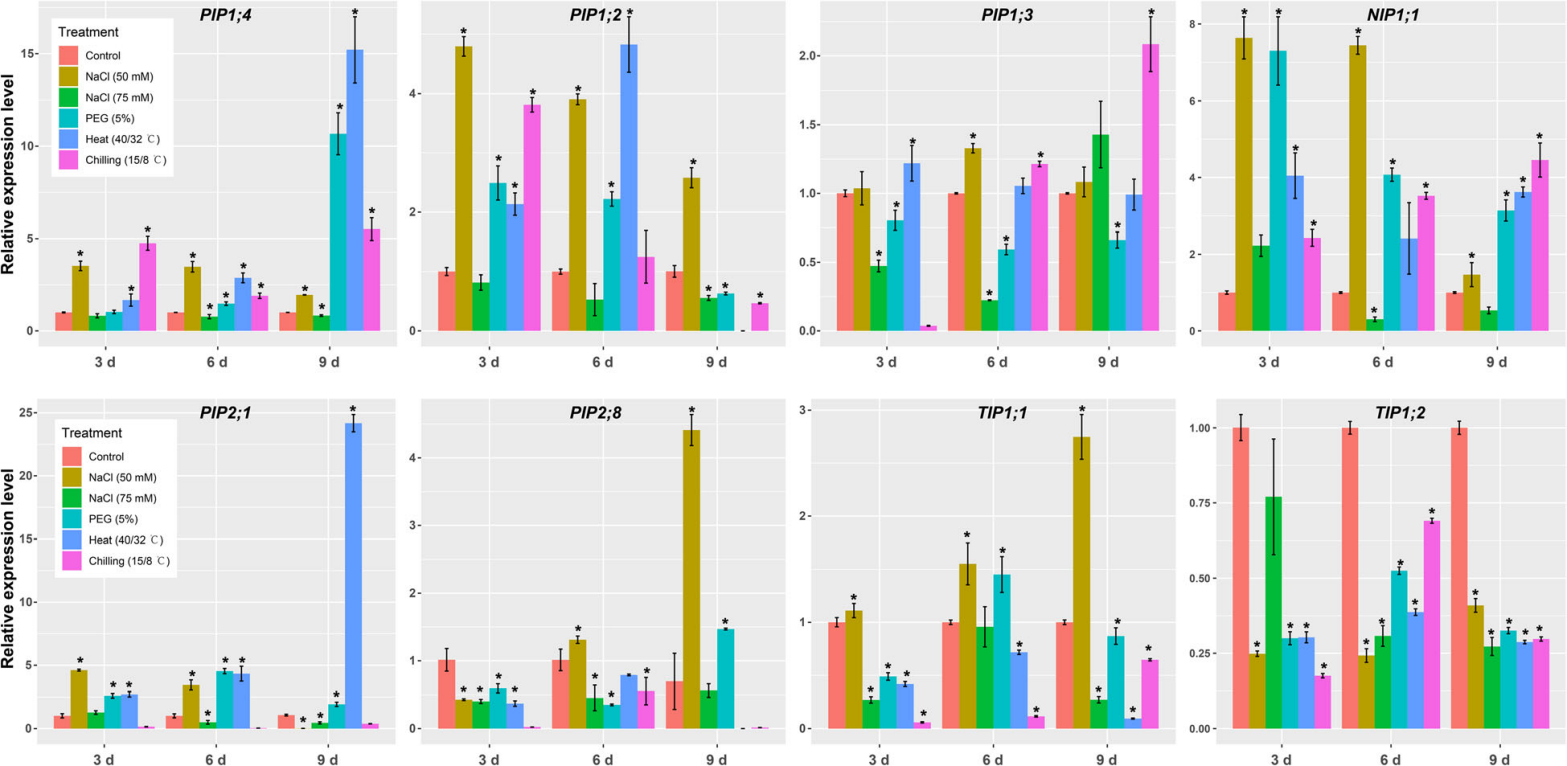
The expression patterns of eight CsAQPs in roots. The roots were sampled after 3, 6, and 9 days of NaCl (50 mmol, 75 mmol), PEG-induced drought, heat, and chilling treatments. The relative expression was determined by qRT-PCR. Values are mean ± SD of three replications, and each replication included two technical replications. * indicate significant differences in comparison with the control at *p* < 0.05, respectively

A number of studies suggested that plant AQPs respond differently to various stress conditions. Accordingly, several highly expressed and/or differentially expressed AQP genes in the root were further analyzed by qRT-PCR (Fig. 10). However, the expression patterns of candidate genes in the cucumber root differ related to salt stress intensity. Most of the AQP genes were down-regulated after 3, 6, and 9 days of 75 mM NaCl treatment, except for *CsPIP1;3*, *CsPIP2;1*, and *CsNIP1;1*, the expression levels of which were increased after 3, 6, and 9 days of treatment, respectively. Consistent with RNA-seq results, *CsTIP1;1*, *CsTIP1;2*, *CsPIP1;3*, *CsPIP2;8* were significantly up-regulated. Moreover, the expression patterns of several genes fluctuated during salt treatment. For example, *CsNIP1;1* was significantly up-regulated after 3 days of salt treatment but down-regulated after salt treatment for 6 or 9 days. *CsTIP1;1* decreased after 3 days of treatment, but returned to control level after 6 days of treatment, and then decreased again. According to RNA-seq results, *CsTIP1;2* and *CsPIP1;4* were significantly down-regulated after 3 days of treatment. Similarly, the expression levels of *CsTIP1;2* and *CsPIP1;4* were decreased according to qRT-PCR although they were not statistically significant at *p* = 0.05. After 6 or 9 days of treatments, *CsTIP1;2* were significantly down-regulated while the expression levels of *CsPIP1;4* were basically unchanged compared with control. However, under 50 mM NaCl stress, the transcriptional levels of most AQP genes increased throughout the NaCl treatment, except for the transcriptional levels of *CsPIP2;1* after 9 days of treatment, *CsPIP2;8* after 3 days of treatment, and *CsTIP1;2* at all time points, the expression levels of which were decreased.

Under PEG (5%)-induced drought stress condition, the expression of *CsPIP1;3,* and *CsTIP1;2* were down-regulated at all time points compare with control. Similarly, the expression levels of *CsPIP2;8, CsTIP1;1*, and *CsPIP2;1* were down-regulated at most time points, except for *CsPIP2;8* after 9 days of treatments, and *CsTIP1;1* and *CsPIP2;1*3 after 3 days of treatments. The expression levels of *CsPIP1;4*, *CsNIP1;1*, *CsPIP2;1* increased throughout the experiment. The expression level of *CsPIP1;2* increased after 3 and 6 days of treatment, but decreased after 9 days of treatment.

Under heat stress, *CsPIP1;4*, *CsNIP1;1*, and *CsPIP2;1* exhibited a significant improvement in expression levels compared with control. The expressions of *CsPIP1;2* and *CsPIP1;3* significantly increased after 3 and 6 days of treatment, but decreased after 9 days of treatment. Compared with control, *CsPIP2;8*, *CsTIP1;1*, and *CsPIP1;3* were downregulated throughout the heat treatment.

In the case of chilling stress, half of the selected AQP genes (*CsPIP2;1*, *CsTIP2;8*, *CsTIP1;1*, and *CsTIP1;2*) were significantly downregulated compared with control, whereas *CsPIP1;4* and *CsNIP1;1* were upregulated throughout the treatment time course. *CsPIP1;2* showed up-regulation at 3 days, but gradually down-regulated thereafter. The expression level of *CsPIP1;3* was down-regulated at the beginning of chilling stress (3 days) but was subsequently up-regulated. These results suggested that stress response of aquaporins is highly variable depending on aquaporin isoform, type and level of stresses.

### Relative water loss rates analysis

**Fig. 11:**
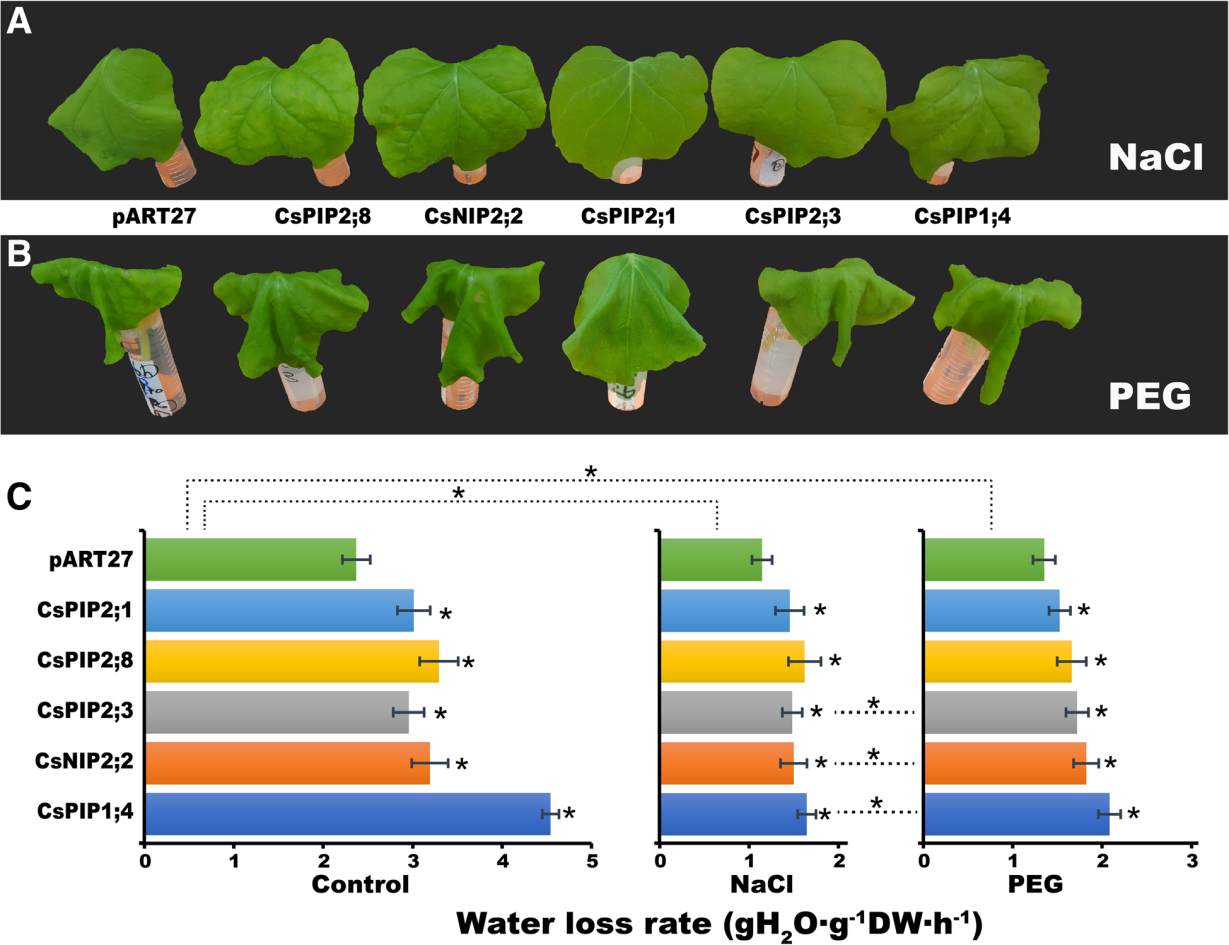
Overexpression of AQPs transiently affected water loss rate in tobacco leaves. Phenotype of detached leaves after 6 h of (**a**) NaCl and (**b**) PEG treatments; (**c)** The water loss rate of detached leaves after 6 h of NaCl, and PEG treatments. In (**c**), asterisks in the left (CK), middle (NaCl), and right (PEG) panels indicate statistically significant differences of each gene compared to pART27. Asterisks above left (CK), middle (NaCl), and right (PEG) panels indicate statistically significant differences of each gene in NaCl or PEG treatment compared to that in CK. Asterisks between middle (NaCl), and right (PEG) panels indicate statistically significant differences of each gene between NaCl and PEG treatments. Data are means ± SE of *n* = 3 biological replicates, at least 10 leaves per replicate. * indicates significance, *p* < 0.05 as determined using the Student’s *t*-test

To evaluate the potential ability of these genes in water transportation, pART27:AQP:GFP fusion constructs (CsPIP1;4, CsNIP2;2, CsPIP2;3, CsPIP2;8, CsPIP2;1) and pART27:GFP (free GFP, used as control) were expressed transiently in the leaves of *N. benthamiana*. As shown in Fig. 11, under CK, NaCl, and PEG-induced drought stress conditions, water loss rate of the detached leaves expressed GFP transiently were lower than that of CsPIP2;1, CsPIP2;8, CsPIP2;3; CsNIP2;2, CsPIP1;4, suggesting that AQP genes increased leaf water loss rate when transiently expressed in tobacco leaves. When compared with CK, NaCl and PEG-induced drought stress significantly decreased water loss rate regardless of types of genes examined, whereas PEG-induced drought stress results in higher water loss rate compared with NaCl treatment.

## Discussion

Genome-wide identification and structure characterizations of AQP gene family. In this study, a genome-wide scan of AQPs was performed for the first time in the cucumber genome and obtained a total of 39 non-redundant AQP genes (Table 1). The number of aquaporins identified in the cucumber is higher compared to species like *Arabidopsis thaliana* (35) [8], Physic nut (32) [17], and bamboo (26) [32], but lower than species like *Brassica oleracea* (67) [33], flax (51) [23], and cotton (71) [9].

Overall, cucumber aquaporins are clustered into five distinct subfamilies: CsPIP (19), CsTIP (8), CsNIP (9), CsSIP (2), and CsXIP (1) (Fig. 1). The XIP subfamily was found to be lost in Brassicaceae species and monocots. In cucumber, CsXIP (1) was less than other dicot species, such as potato (8) [10], and tomato (6) [25]. Alternative splicing (AS) events have been identified in PIPs (*CsPIP2;1*, *CsPIP2;3*, *CsPIP2;4*, *CsPIP2;6*) and NIPs (*CsNIP3;1*), which resulted in the production of isoform transcripts (Fig. 4b and Additional file 1: Table S1), suggesting a more complex nature of cucumber aquaporins. Some of the CsAQPs were found to encode truncated proteins that lack one (CsPIP1;2b, CsPIP2;1b, and CsPIP2;3a, and CsNIP3;1b) or two (CsPIP1;5, CsNIP1;1) transmembrane helical domains (Table 1). The absence of transmembrane helical domain(s) may affect subcellular localization and water transport activity. For example, tdpip2;1 (a truncated form of wheat TdPIP2;1 aquaporin) had no water channel activity. Nonetheless, the functional TdPIP2;1 can potentially interact with the truncated tdpip2;1, allowing it to reach the plasma membrane, where tdpip2;1 may compete with the functional form and thus disrupt the activity of aquaporin [34].

### PIPs and TIPs

The function of aquaporins in regulating water flow across biological membranes and in maintaining cellular water homeostasis are well known [30]. PIPs and TIPs are the most abundant and extensively studied AQPs under both normal and stress conditions, because they have higher water transport activity compared to NIPs and SIPs [35]. However, information on structural features and evidence of their functions in response to salt stress are largely unknown in cucumber. CsPIPs were divided into two subgroups and CsTIPs were divided into CsTIP1 to CsTIP5 (Table 1). The relative conservation of NPA motifs and ar/R residues between PIPs and TIPs members of cucumber and other species suggested a conserved function for these proteins in transporting water and small neutral solutes, such as CO_2_, H_2_O_2_ for PIPs, and NH_4_^+^, H_2_O_2_, urea for TIPs (Table 1) [5]. A general down-regulation of most PIP and/or TIP genes is thought to reduce water loss under stress (e.g. salt and drought) conditions, through affecting whole-plant hydraulics [28, 30, 36]. For example, in Arabidopsis, a down-regulation of the most abundant PIP and TIP aquaporin transcripts (60 to 75% respectively) was observed after exposure to salt stress, coupled with a significant reduction in root hydraulic conductance [37]. Although the contribution of aquaporins to leaf hydraulic conductance was much less than that in the root, Arabidopsis PIPs can account for a significant portion of aquaporin-mediated leaf water transport [38]. Similarly, in salt-tolerant tomato species, a higher basal expression of PIP2;1 largely contributed to its better hydric status under salinity [39]. Among few experiments related to the cucumber AQPs, Qian et al. [40] found that several highly expressed cucumber PIPs may have a great contribution to the reduction of hydraulic conductivities. Our previous study also found that higher basal expression of root PIP aquaporins (especially *CsPIP1;2* and *CsPIP2;4*) in silicon-treated cucumber could be one of the causes of its improved hydric status under salinity [28].

Expression profiling using previously released RNA-seq data (http://cucurbitgenomics.org/organism/2) showed that 10 CsAOPs including 5 CsPIPs accounted for about 50% of the total expression of AOPs in different tissues. *CsTIP1;1*, *CsPIP1;2*, *CsPIP2;4*, and *CsPIP2;8* showed high expression levels in almost all tissues (except for seeds) and different growth stages, suggesting their possible role in these tissues (Fig. 7). Moreover, the expression levels of these genes are differentially affected by various treatment including silicon and powdery mildew. In order to further elucidate their response pattern to salt stress, RNA-seq was performed to examine the transcriptomes of cucumber root under salt stress conditions. Similar to analysis results of public RNA-seq data, *CsPIP1;2*, *CsPIP1;3*, *CsPIP2;4*, *CsPIP2;8*, and *CsTIP1;1* were among the most abundant aquaporins in the root of cucumber, accounting for 80% of the total expressed AQPs. Moreover, expression levels of these highly abundant AQPs were significantly decreased by salt stress, which may account for the down-regulation of hydraulic conductance response to salt stress (Fig. 10).

Although it is hard to identify a concerted expression pattern of all AQPs in response to salt stress on the bases of transcriptome data, we suggest that PIPs and TIPs, especially three aquaporins, *CsPIP1;2*, *CsPIP2;4*, and *CsTIP1;1*, which are highly expressed and their expression level were significantly altered by salt stress, may play a central physiological role in regulating water movement and homeostasis in cucumber. Further functional analyses of these AQPs are necessary to understand the roles of individual AQP members as well as reveal their structural features that are crucial for physiological substrate transport.

### NIPs

Nodulin-26-like intrinsic proteins (NIPs) subfamily encompasses a high sequence diversity, and NIPs facilitate the transport of the widest range of solutes, including small organic solutes and mineral nutrients [21]. In Arabidopsis and rice, NIP families contained 7 and 4 NIP subgroups respectively [8, 41], whereas analysis of the cucumber NIPs showed the presence of four phylogenetic groups. The ar/R selectivity filter of CsNIP1 group is typical of the subgroup I of plant NIP aquaporins, with the residues Tryptophan/Valine/Alanine/Arginine (W/V/A/R) in the ar/R filter (Table 1). Due to these structural similarities, CsNIP1;1 and CsNIP1;2 may have similar transport specificity as formerly demonstrated in subgroup I of other plant NIPs, including low water permeability, and capable of transporting uncharged solutes like formamide and glycerol [21].

NIP2 subgroup is the only aquaporin subgroup able to transport silicon (Si). Si is the second most prevalent element in the soil, and has been reported to be beneficial in alleviating various environmental stresses [1, 3]. Rice Lsi1 (OsNIP2;1) is the first silicon transport protein identified in plants. The ar/R region of OsLsi1 consists of four small-sized residues, glycine, serine, glycine, and arginine (GSGR), that form a larger constriction pore, allowing relatively large molecules of silicic acid to permeate through [42]. In cucumber, Sun et al. [43] isolated and characterized *CsLsi1*, a gene encoding a silicon influx transporter. In this study, *CsLsi1* was systematically classified and named as *CsNIP2;1* based on phylogenetic analysis. Similar to rice *OsNIP2;1*, *CsNIP2;1* has a signature sequence with the GSGR ar/R selectivity filter. Deshmukh et al. [44] proposed that a spacing of a specific length (108 AA, amino acids) between the two NPA domains is a necessary and selective feature for Si among Si-transporting plants. For example, 108 amino acids were found in rice, sorghum, sunflower, and purple false brome, which accumulate more than 3.5% silicon in the leaf on the basis of dry weight [45]. While in tomato, lower silicon content ranging from 0.2 to 0.7% (on the basis of leaf dry weight) alone with a spacing of 109 amino acids between the two NPA domains were reported [44, 46]. In cucumber, a dicot with a relatively high capacity for silicon accumulation [47], 107 amino acids between the two NPA domains was detected, with the values for cv. Xinyan No.7 and cv. Jinyou No.1 in the shoot and leaves being 1.4 and 1.1% Si, respectively (see Wu et al. [46] and Additional file 2: Figure S4). These results suggested that AQPs with specific characteristics could be an important molecular basis for Si permeability in plants as proposed by Deshmukh et al.[44]. However, field data should also be taken in to consideration since silicon transporter genes that not do belong to AQPs family like CsLsi2 (Csa3G182780.1) [47], which functions to transport silicon out of the endodermal cells into the stele for xylem loading, may also largely influence silicon content in shoots.

### XIPs

XIPs have been encoded by the genomes of some higher plants, such as tomato [25], poplar [11] and common bean (*Phaseolus vulgaris* L.) [48]. In several Solanaceae species, XIPs showed negligible water permeability while facilitated boric acid, urea, and H_2_O_2_ transport compared to other AQP subfamily. Generally, *CsXIP1;1* showed low expression levels in different cucumber tissues, and no expression in the seeds. Moreover, *CsXIP1;1* was largely up-regulated or down-regulated when infected with downy mildew or subjected to salt stress, respectively (Fig. 7). Only a few experiments have implied the involvement of XIPs in salt stress. Transcriptome analyses of sweet orange (*Citrus sinensis* L. Osb.) under salt stress revealed that XIPs are up-regulated in roots, whereas in leaves both up and down regulation patterns were observed [49]. Further studies are required to explore the transported substance of CsXIP1, and quantify its precise expression levels in different organs and in response to different environmental conditions, which will help us to elucidate its physiological significance in cucumber.

#### RNA transcript profiling

Experiments in several plant species demonstrated that AQPs showed differential expression patterns in response to environmental stresses, such as drought, and salinity [20]. Regarding cucumber, functional studies on cucumber aquaporin are still lag behind; expression profile analysis of aquaporin may provide useful information for establishing their putative functions. Heatmap constructed from previous transcriptomic studies have provided a better understanding of the transcript abundance pattern of CsAQPs in different tissues and under various treatments. We found that the expression pattern of aquaporins was highly variable depending on aquaporin individual, tissue, species, stress type, levels and duration (Fig. 7; Additional file 2: Figure S2).

#### Salt stress decreases root water uptake and hydraulic conductivity

Salinity stress is the combination of water and ionic stress that bring about deleterious effects on several major processes in plants [1]. One of the primary responses of plants to salinity stress is the inhibition of root water uptake and a resultant decrease in root hydraulic conductivity. In this study, salt stress significantly decreased cucumber water content and root hydraulic conductivity after 3 days of treatment (Fig. 8a, b). Water and ionic stress, initially sensed by the roots, can affect the flow of water into the leaf, which lowers the leaf water potential and then leaf hydraulic conductance of cucumber (Fig. 8c–h) [50]. Root and leaf hydraulic conductivity reflect the water uptake and transport capacity of plant. In many plant species, the regulation of plant hydraulic conductivity in response to environmental stresses are primary mediated by aquaporins [36, 51]. For instance, in Arabidopsis, salt treatment caused a significant reduction in root hydraulic conductivity, coupled with a 60 to 75% decrease in PIP and TIP transcripts abundance [37]. Moreover, an abundant plasma membrane aquaporin, AtPIP1;2, can account for a significant portion of aquaporin-mediated leaf water transport [38]. In present study, the decreased leaf and root hydraulic conductivity and water content of salt-stressed cucumber may be largely due to the downregulation of specific aquaporin genes, just as suggested by our previous study [28]. Similarly, Qian et al. [40] had identified 10 PIP genes based on the cucumber genome v1 [52], and proposed that two highly expressed cucumber PIPs, *CsPIP1;2* (Csa5G198770) and *CsPIP2;4* (Csa6G140850), greatly contribute to the reduction of hydraulic conductivities in salt and drought-stressed cucumber. Since then, cucumber genome sequencing has been further completed (http://cucurbitgenomics.org/organism/2), but a comprehensive study of AQP genes in cucumber is lacking. Moreover, the relationship between cucumber AQPs and salt stress is largely unknown.

Furthermore, transcriptome profiling analysis was carried out in this study to identify CsAQPs that respond to salt stress. Most root AQP genes were greatly down-regulated during NaCl treatment (Fig. 9). And these down-regulated AQP genes may contribute greatly to salt-decreased water content and root hydraulic conductance (Fig. 8). Few low expression AQP genes displayed up-regulation expression after 3 days of salt treatment, nevertheless, these lowly expressed AQP genes may have minor contribution to water uptake capacity of plant roots, which could not counteract the lower expression of other AQP genes. H_2_O_2_ was found to play a negative role in regulating the activity of aquaporin in cell and/or root water conductivity [53]. In *Sorghum bicolor*, it has been observed that maintenance of aquaporin activity and increased root hydraulic conductance may be partly due to the decreased H_2_O_2_ levels caused by Si treatment [53]. Similarly, in this study, salt-induced H_2_O_2_ accumulation may affect roots aquaporin to some extent, and thus root hydraulic conductance, but this needs further investigation.

Unlike in roots, the expression patterns of CsPIP genes in leaves changed in different ways in response to NaCl treatment, with a down-regulation of *CsTP1;1* and *CsPIP2;8*, and an up-regulation of *CsTIP1;1* and *CsPIP2;8*. These were the top four highest expressed AQPs in leaf tissue making up more than 50% transcript abundance of all AQPs. The variable expression patterns of PIP genes in leaf tissue under stress condition were also observed in another cucumber cultivar (*C. sativus* L. “Yuexiu 3”) [40], rice [54] and *Arabidopsis thaliana* [55]. Qian et al. [40] proposed that the down-regulation of the two most highly expressed CsPIPs (CsPIP1;2 and CsPIP2;4), may contribute highly to the reduction of hydraulic conductivities both at the whole leaf and cell level. In this study, highly variable leaf AQPs expression levels was an unlikely explanation for the decreased hydraulic conductivity upon exposure to salt stress conditions. Similarly, inconsistency between aquaporin expression at the mRNA level and protein accumulation have been noted in previous studies, although the expression levels of specific aquaporin genes are important to control water transport in plants. For example, the translation rate and degradation of PIP proteins could differentially influence PIP protein levels independently from transcription levels [54]; Moreover, tissue hydraulic conductivity could also be regulated by other processes including membrane insertion and stability [55]. Thus, this diversity of AQPs expression patterns in the cucumber leaf tissues implies that the role of aquaporins in salt resistance is influenced by many factors and the discrepancy between leaf AQPs expression level and leaf hydraulic conductivity need to be further explored.

More complete analyses of the responses of *CsAQP* genes to different salt concentrations and abiotic stress treatments will help to better understand their effects on stress-related physiological processes and eventually allow for elucidation of the contribution of aquaporins to overall water movement. We then used qRT-PCR analysis to elucidate the responses of *CsAQP* genes to different salt concentrations, PEG-induced drought stress, heat, and chilling stresses, and results showed that selected eight *CsAQP* genes are expressed differently upon various abiotic stress treatments. Previous studies suggested that plants responded differentially to different NaCl concentrations and stress duration. For example, in maize, three highly expressed PIPs (*ZmPIP1;1*, *ZmPIP1;5*, and *ZmPIP2;4*) were transiently induced after 2 h of 100 mM NaCl treatment. By contrast, multiple ZmPIP and ZmTIP genes were repressed after 24 h of 200 mM NaCl treatment. Similarly, differential responses of *CsAQP*s were observed after adding 50 mM (mild salt stress) or 75 mM NaCl (moderate salt stress). Generally, these eight *CsAQP* genes were more highly induced in response to 50 mM NaCl than 75 mM NaCl. The comparison of the effects between different NaCl concentrations on the *CsAQP*s genes revealed differential and gene-specific effects.

Similar to salt stress, drought, heat and chilling stresses are important abiotic stress factors that have great impacts on plant growth and development [56–58]. Evidence shows that AQPs were involved in these stress responses in plants. Both drought and salt treatment could induce osmotic stresses that affect plant water balance. In consistent with this, many experimental evidences suggested that aquaporin responses in salinity is in consistent with drought stress [30]. However, other experiments reveal opposite patterns. In grapevine, Cramer et al. [59] reported that the expression of *PIP2;1* gene is down-regulated under drought but up-regulated under salt stress. In cucumber, 2 h PEG and NaCl treatments significantly increased the transcript levels of most *CsPIP* genes in roots, whereas 24 h PEG and NaCl exposure significantly decreased and increased mRNA levels of most *CsPIPs*, respectively [40]. In this study, under PEG-induced drought stresses, CsAQPs genes showed variation in transcript at different time points compared to the control, suggesting differential regulation of CsAQPs by drought and salt stress. Interestingly, similar alteration patterns were seen in eight selected CsAQPs in response to heat and drought stress, and differences were mainly focused on the degree of expression of *CsAQP*. In the case of chilling stress, differential expression of eight aquaporin homologs suggested definite roles in stress responses. These results indicated that the expression profile of *CsAQPs* genes may be dependent on stress types, intensity, and duration. As the next step, we should quantify the contribution of each aquaporin isoforms in the complex stress-response process.

In many studies, transgenic plants overexpressing aquaporins showed enhanced drought and salt tolerance through enhancing water retention capabilities [30]. For example, the overexpression of *MaPIP1;1* (a banana PIP gene) in Arabidopsis decreased plants water loss rate and maintained higher levels of proline and osmotic potential compared to wild type plants [60]. Wang et al. [14] found that ectopically expressing apple *MdPIP1;3* enhanced drought tolerance of transgenic tomatoes partially through reducing leaf water loss controlled by stomata closure. However, several contrasting results have also been reported because increased leaf and root hydraulic conductivity result in rapid water loss, which makes some plants even more vulnerable to drought stress conditions. Aharon [61] found that overexpression of Arabidopsis PIP1b in transgenic tobacco plants facilitate water loss due to increased leaf and root hydraulic conductivity, which makes plants more vulnerable to drought stress. Similarly, Jang et al. [62] reported that Arabidopsis and tobacco plants overexpressing Arabidopsis PIP1;4 or PIP2;5 showed a rapid water loss under drought stress, which hindered germination and seedling growth under dehydration stress. In this study, transient expression of these CsAPQs in tobacco displayed higher water loss rate compared to control GFP under both salt and drought conditions, suggesting a role of CsAPQs in water transportation. However, it is hard to decide the overall behavior of AQP in plants under stress conditions. Firstly, root water absorption capacity and the whole plant hydraulic conductance could not be estimated through solely expression of these CsAPQs transiently in tobacco leaves. Secondly, AQPs have other physiological functions other than water transport ability. For instance, AQPs have been proposed to participate in improving ion distribution, maintaining osmotic balance and improving carbon bioavailability, which also alter the resistance of the plants to stresses conditions [63]. Further studies are required to characterize the functions of these AQPs that constitutively overexpress in cucumber under various abiotic stress conditions.

## Conclusions

A total of 39 AQP genes in five sub-families were identified and characterized based on their sequences, phylogenetic relationships, genomic organization, tissue-specific gene expression, and expression profiles upon abiotic and biotic stresses. The RNA-seq data revealed several generally highly expressed AQPs including *CsTIP1;1*, *CsPIP1;2*, *CsPIP2;4*, and *CsPIP1;3*, which showed higher abundance in almost every tissue except in the seed, suggesting the important roles of these AQPs. Several CsAQPs (*CsTIP1;1*, *CsPIP1;2*, *CsPIP2;4*, *CsPIP1;3*) were found to be highly expressed in ovary, fruits and/or flower, indicating a role transporting water or solutes in fruit development. Moreover, AQPs in the root and leaf respond differentially to salt stress. The expression of root AQPs was decreased by salt treatment, which may be due largely to increased H_2_O_2_ content induced by salt stress, and resulted in decreased root hydraulic conductivity. qRT-PCR analysis showed that diverse abiotic stresses could alter the expression levels of CsAQPs. Moreover, transient expression analysis indicated that AQPs play roles in the regulation of plant water status. Under stress conditions. Further molecular study of CsAQPs should reveal more functional mechanisms for these genes. These results can also further expand our understanding of the AQPs in cucumber and may contribute to genetic engineering for cucumber stress-resistance improvement.

## Methods

### Identification of AQP genes in cucumber

The whole genome sequences of cucumber were downloaded from Cucurbit Genomics Database (http://cucurbitgenomics.org/organism/2). The protein sequences of AQPs in the model plants Arabidopsis (http://www.arabidopsis.org/index.jsp) [8], rice (http://rice.plantbiology.msu.edu/index.shtml) [64], maize (https://www.maizegdb.org/) [65], and potato [10] were used as queries to carry out local BLASTp searches in the Cucurbit Genomics Database with a cutoff e-value of 1e-10. Furthermore, the keyword “aquaporin” was used as query to search the Cucurbit Genomics Database. Retrieved sequences were submitted to InterProScan (http://www.ebi.ac.uk/interpro/) to verify the AQP domains (IPR015686, IPR016697, IPR023265, IPR023266, IPR023271, IPR023274, IPR023276, IPR023743, IPR026252, and IPR031145). Since no XIP gene (one of the five aquaporin sub-families) was detected in Arabidopsis, rice, maize, or potato, XIPs were collected from *Ricinus communis*, *Hevea brasiliensis*, and *Hevea brasiliensis* and used to detect potential cucumber XIPs (see reference in [17]). The AQP protein sequences were further examined to confirm the presence of the characteristic MIP and trans-membrane helical domains using the SMART program (http://smart.emblheidelberg.de/) [66] and TMHMM (http://www.cbs.dtu.dk/services/TMHMM/) [67].

### Sequence alignments, phylogenetic analysis, and classification of cucumber aquaporins

Phylogenetic analysis was used to classify AQPs into subfamilies. Aquaporin amino acid sequences from cucumber, Arabidopsis, rice, maize, and potato, and XIPs from other plants were aligned using ClustalW2 software. An unrooted phylogenetic tree was constructed using MEGA7 with the neighbor-joining method based on the LG model, and 1000 bootstrap test replicates were used during the construction [68]. The phylogenetic tree was illustrated using Interactive Tree of Life (IToL, v3.2.317, http://itol.embl.de). The combined tree was generated to systematically classify AQPs and the systematic names were assigned based on their evolutionary relationships. The aligned sequences were used to identify conserved regions present in AQP sequences and to analyze the ar/R contents and Froger’s positions.

### Characterization of aquaporin gene structures and protein transmembrane structures

Gene annotations of the identified cucumber aquaporins were extracted from the genome reference GFF file (Additional file 1: Table S1). The start and end location information of these aquaporins in chromosomes were used to draw a physical map using the software MapInspect (http://mapinspect.software.informer.com). The conserved motifs of cucumber AQPs were identified using MEME motif search tool (http://mem-esuite.org/tools/meme) [69]. Default parameters were used in this study, except that the maximum number of motifs was set to 20. The motif patterns were drawn using TBtools software (https://github.com/CJ-Chen/TBtools). Additionally, the online tool ProtParam (https://web.expasy.org/protparam/) was used to predict isoelectric point (PI), relative molecular weight (MW), instability index, atomic composition, and amino acid composition. The subcellular localization of AQP proteins was predicted using the WoLF PSORT tool available at http://www.genscript.com/psort/wolf_psort.html.

### *Cis*-elements and homology modelling analysis

Custom Perl script was used to extract the 2 kb upstream region of genes. PlantCARE database was used for the identification of *cis*-regulatory elements in the promoter regions. The Phyre2 server was used to homology modelling the three dimension structure of cucumber aquaporins.

### RNA-seq data analysis of AQP genes

To explore the expression patterns of AQP genes, the expression level of each AQP represented by FPKM values (Fragments Per Kilobase of transcript per Million fragments mapped values) was collected from the Cucurbit Genomics Database (http://cucurbitgenomics.org/organism/2). The RNA-seq data included a wide range of developmental stages and multiple growth conditions. The R package “pheatmap” was used to draw the heatmap of cucumber AQP genes based on their expression levels. Boxplot was used to illustrate the overall expression patterns of AQP genes. Based on the expression patterns, correlation of AQP genes were analyzed by R function “cor” and were illustrated by R package “heatmap”.

### Plant material and treatment

Cucumber (*Cucumis sativus* L. “JinYou 1”, Xintiandi Co., Yangling, Shannxi, China) seeds were surface sterilized in 55 °C hot water for 10 min and germinated at 28 °C for 2 days. Then, germinated seeds were sown in trays containing sand, perlite, and peat in a 1:1:1 ratio and incubated in a growth chamber (relative humidity of 60–70% with a 12 h light period and 28 °C/18 °C day/night temperature). The two-leaf stage seedlings of uniform size were transferred to 30-L plastic containers filled with continuously aerated 1/2 Hoagland nutrient solution, and each container had 15 plants. Seven days after transplanting, four experimental groups were designed: (i) Control, seedling were grown in 1/2 Hoagland nutrient solution (28 °C/18 °C day/night temperature); (ii) Salt stress, sodium chloride (NaCl 50 and 75 mM) were added to the nutrient solution; (iii) Heat stress, seedling were grown at 40 °C/32 °C day/night temperature; (iv) Chilling stress, seedling were grown at 18 °C/5 °C day/night temperature. The pH of nutrient solution was adjusted to 6.0 using 0.2 M H_2_SO_4_ or 1 M KOH every day. The leaves and roots were collected after 3, 6, and 9 days of treatment. All sampled materials were quickly frozen in liquid nitrogen and stored at − 80 °C prior to RNA extraction.

### Plant biomass, water status, and H_2_O_2_ content

After 3 days of treatment, cucumber seedlings were harvested. The shoots and roots were separated and quickly washed in double-distilled water and then placed in an oven at 70 °C for 48 h [28]. Total water content was measured according to [70]. H_2_O_2_ content were assayed according to the method of Yin et al. [1] after 3 days of treatment.

### Measurements of leaf and root hydraulic conductivity and leaf water potential

The leaf-specific conductivity of the stem was measured using pressure chamber technique [71]. The leaf-specific conductivity was calculated as: L_sc_ = F × SL/ΔP/A_leaf_ (kg H_2_O s^− 1^m^− 1^MPa^− 1^), where F represents for the flow rate (kg s^− 1^), SL represents for the stem length, ΔP is the hydrostatic gradient and expressed in MPa, and A_leaf_ means the “supported leaf area” of each segment. Root hydraulic conductance (Lp_r_) were measured and calculated according to Zhu et al. [28]. Leaf water potential was determined between 10:00 and 11:00 AM on the second fully expanded young leaf, using a pressure chamber [40].

### RNA extraction and Illumina sequencing

Three days after salt treatment, roots were collected and frozen in liquid nitrogen immediately and stored at − 80 °C prior to the assays. Three independent biological samples were used in the analysis to ensure the accuracy of the analysis. RNA-Seq library construction and sequencing were conducted by Gene Denovo Co. (Guangzhou, China) using an Illumina HiSeq 2500 platform. After filtering, high quality reads were mapped onto the reference genome of cucumber (v2, http://cucurbitgenomics.org/organism/2) using TopHat2 [72]. The expression levels of the cucumber annotated genes across all samples were normalized to a baseline read count of one million reads using Trimmed Mean of M-values normalization algorithm in the edgeR package [73]. EdgeR was used to identify the differentially expressed genes (DEGs). A threshold of false discovery rate (FDR) ≤ 0.05 and an absolute value of log2 (Ratio) ≥ 1 were used to set the difference significance of DEGs. The sequencing reads of root materials produced by this project have been submitted to the National Center for Biotechnology Information (NCBI) Sequence Read Archive with a Bioproject ID of PRJNA511946. And sequencing reads of leaf materials, produced by our previously project with accession number of GSE116265, were also used in this study.

### qRT-PCR analysis

The cucumber roots collected from plants treated with NaCl (50 and 100 mM), heat, polyethylene glycol (PEG)-6000 and chilling for 3, 6 and 9 days and were frozen in liquid nitrogen for RNA extraction. The first-strand cDNA templates were synthesized from 1 μg total RNA using PrimeScritpt RT reagent Kit with the Vazyme RT-PCR system in a total volume of 25 μl according to the manufacturer’s instructions (Vazyme, Nanjing, China). qRT-PCR assays were conducted on each cDNA sample using SYBR Green Master Mix (Vazyme, Nanjing, China) on a CFX 96 Real-Time PCR system (Bio-Rad) with CsAQP gene-specific primers (Additional file 1: Table S2). The relative expression levels were calculated using the 2^-△△ct^ method [74]. Each treatment included three biological replicates with two technical replicates.

### Subcellular localization analysis of AQP proteins

To confirm the sub-cellular localization of CsAQPs predicted using the online tool WoLF PSORT tool, the AQP:GFP fusion constructs were constructed. Briefly, total RNA of cucumber was extracted using Plant RNA kit (Omega, London, UK) and cDNA was synthesized using RevertAid First Strand cDNA Synthesis Kit (Thermos Scientific, Madison, WI, USA). Full length of CsAQPs were amplified by Phanta HS Master Mix (Vazyme, Nanjing, China). Primers were synthesized by Sangon Biotech (Shanghai, China) as listed in Additional file 1: Table S3. The vector pART27:GFP was digested with *Xho*I (NEB, Nanjing, China) and then purified by Cycle-pure Kit (Omega, London, UK). PCR products were inserted into lined pART27:GFP using ClonExpress II One Step Cloning Kit (Vazyme, Nanjing, China). These positive clones were transformed into *Agrobacterium tumefaciens* strain GV3101. The pART27:GFP transformed Agrobacterium (constitutive expressing free GFP) was used as control. Tobacco leaves were transfected by infiltration using an *A. tumefaciens* method. Two days later, the injected leaves were placed on the glass slides and visualized through laser confocal microscopy (Olympus FV3000, Tokyo, Japan). The exciting light wavelength was 488 nm.

### Relative water loss rate analysis

After transient expression pART27:GFP or pART27:AQP:GFP fusions in *N*. *benthamiana* for 2 days, leaves were excised from the plants. The petioles were inserted into centrifuge tube sealed with sponge, which were filled with sodium chloride solutions (400 mM) or PEG solution (1%). Relative water loss rates were determined by short-time weight loss of leaves under laboratory condition of 25 °C and the relative humidity in the greenhouse was 60% [75]. Samples were weighted hourly for 6 h and data were expressed on a dry weight basis (g H_2_O·g^− 1^DW·h^− 1^).

## Additional files

Additional file 1: **Table S1.** GFF3 formatted gene annotations of cucumber aquaporins. **Table S2.** List of primers used for qRT-PCR. **Table S3.** List of primers used for gene cloning. **Table S4.** Sequence similarity matrix of cucumber aquaporins. **Table S5.** Detailed information of *cis*-elements in cucumber aquaporin genes. **Table S6.** Multiple released RNA-seq data used for cucumber aquaporins expression profiling in heatmap. **Table S7.** Summary of cucumber root sequence assembly after Illumina sequencing. **Table S8.** Cucumber roots gene expression levels represented by FPKM. (XLSX 2681 kb)

Additional file 2: **Figure S1.** Maximum likelihood (ML) phylogenetic analysis of the cucumber aquaporin family with members of other plants. **Figure S2.** Transcripts abundance of aquaporin genes. **Figure S3.** Co-expression heatmap based on Pearson’s correlation coefficient. **Figure S4.** Silicon concentration in cucumber seedlings. (DOCX 1331 kb)

## Data Availability

We have deposited our data in the National Center for Biotechnology Information (NCBI) Sequence Read Archive with a Bioproject ID: PRJNA511946.

## Abbreviations

A: Alanine
ABRE: ABA responsive element
AQP: Aquaporin
ar/R: Aromatic/Arginine
ARE: Anaerobic induction regulatory element
CGTCA-motif: MeJA-responsive element
CO_2_: Carbon dioxide
CsAQPs: Cucumber AQP genes
DEGs: Differentially expressed genes
ER: Endoplasmic reticulum
FDR: False discovery rate
FPKM: Fragments Per Kilobase of transcript per Million fragments mapped values
G-box: Light responsive element
GFP: Green fluorescent protein
GRAVY: Grand average hydrophobicity
H: Histidine
H_2_O_2_: Hydrogen peroxide
I: Isoleucine
L: Leucine
LTR: Low-temperature responsive element
MBS: MYB binding site involved in drought-inducibility
MIPs: Membrane intrinsic proteins
NCBI: National Center for Biotechnology Information
NH3: Ammonia
NIPs: Nodulin-26-like intrinsic proteins
NO: Nitric oxide
NPA: Asn-Pro-Ala
PEG: Polyethylene glycol
pI: Isoelectric point
PIPs: Plasma membrane intrinsic proteins
qRT-PCR: Quantitative real time PCR
R: Arginine
S: Serine
SIPs: Small basic intrinsic proteins
T: Threonine
TCA-element: Salicylic acid responsive element
TC-rich repeats: Defense and stress responsive element
TGA-element: Auxin-responsive element
TIPs: Tonoplast intrinsic proteins
TMHs: Transmembrane helices
V: Valine
W: Tryptophan
XIPs: X intrinsic proteins
Y: Tyrosine
