# Supplementary material for: Genome-wide identification, structure characterization, and expression pattern profiling of aquaporin gene family in cucumber
Source: BMC Plant Biol. 2019 Aug 7;19:345. doi: 10.1186/s12870-019-1953-1 (PMC6686268; doi:10.1186/s12870-019-1953-1)
Supplement: Supplementary file 2 — Figure S1. Maximum likelihood (ML) phylogenetic analysis of the cucumber aquaporin family with members of other plants. Figure S2. Transcripts abundance of aquaporin genes. Figure S3. Co-expression heatmap based on Pearson’s correlation coefficient. Figure S4. Silicon concentration in cucumber seedlings. (DOCX 1331 kb) [file 12870_2019_1953_MOESM2_ESM.docx]

**
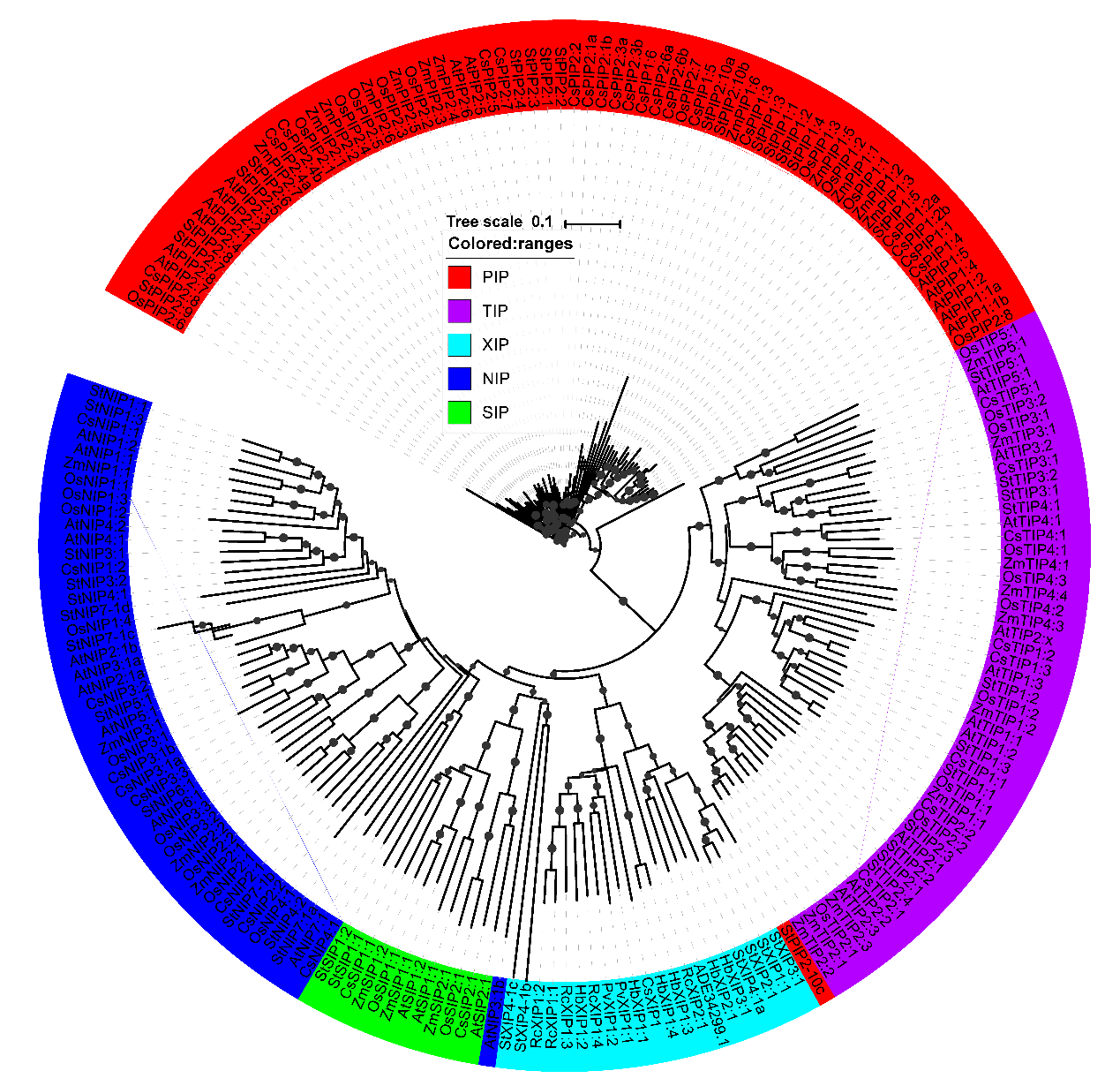
**

**Additional file 2: Figure S1. Maximum likelihood (ML) phylogenetic analysis of the cucumber aquaporin family with members of other plants**. Predicted amino acid sequences were aligned by means of ClustalW2 sequence alignment program and a phylogenetic tree was designed by MEGA7.0 software though Bootstrap NJ tree (1000 replicates) method. The name of each group and subgroup is indicated next to the corresponding group. The distance scale denotes the number of amino acid substitutions per site. At, *Arabidopsis thaliana*; Os, *Oryza sativa*; St, *Solanum tuberosum*; Zm, *Zea mays*; Rc, *Ricinus communis*; Hb, *Hevea brasiliensis*.


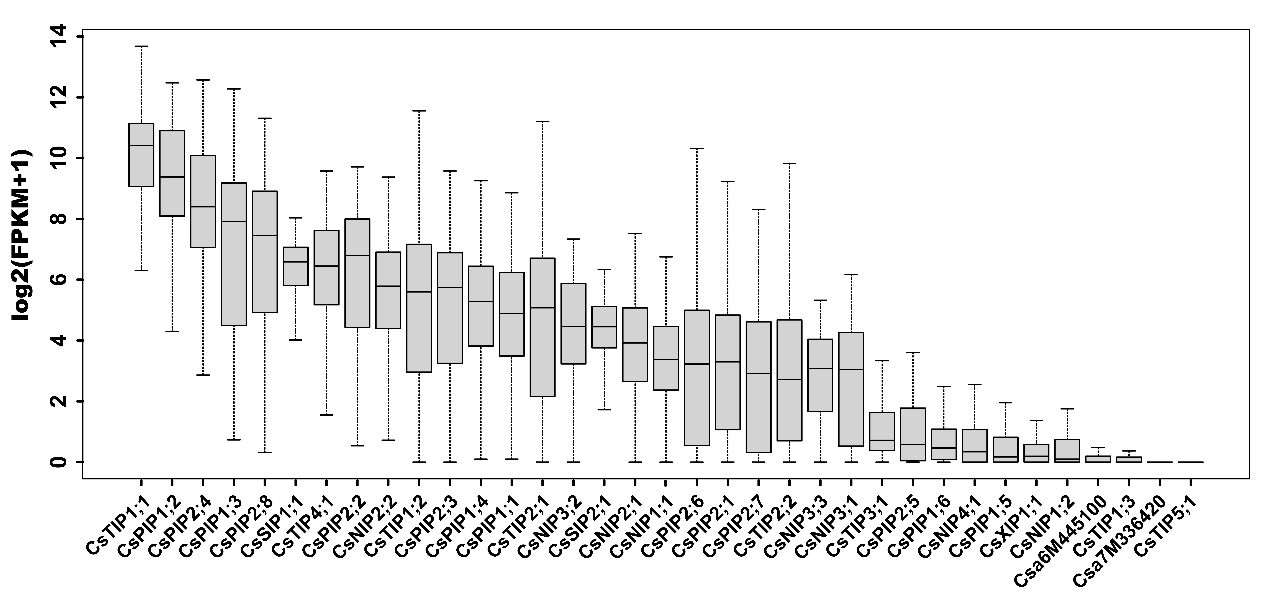


**Additional file 2: Figure S2. Transcripts abundance of aquaporin genes.** The value is calculated using the FPKM values of aquaporin genes which were mined from multiple RNA-seq data including different tissues, development stages, and treatment conditions. All detailed information can be found in Cucurbit Genomics Database (http://cucurbitgenomics.org/organism/2).

**
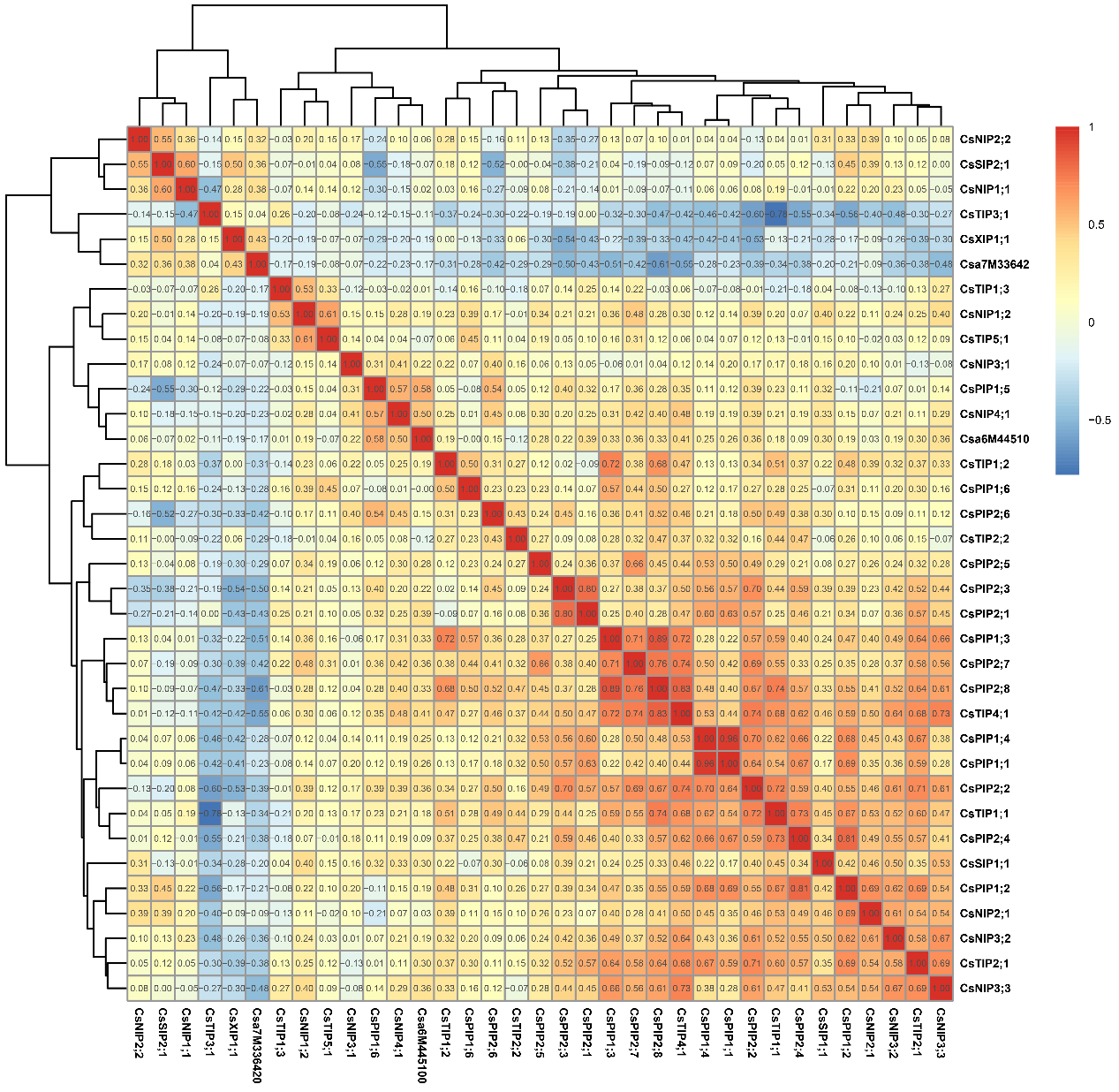
**

**Additional file 2: Figure S3. Co-expression heatmap based on Pearson’s correlation coefficient.** The value is calculated using the FPKM values of aquaporin genes which were mined from multiple RNA-seq data including different tissues, development stages, and treatment conditions. All detailed information can be found in Cucurbit Genomics Database (http://cucurbitgenomics.org/organism/2). Putative interacting aquaporins are with a Pearson’s correlation coefficient (r) > 0.8.





**Additional file 2: Figure S4. Silicon concentration in cucumber seedlings.** Data are mean±SD of five replicates. DW, dry weight. Silicon concentration was determined according to Wu et al (2016).
